# *In Silico* Mining of NPACT Database Toward Identification of EBNA1 Inhibitor: Virtual Screening, Molecular Dynamics Simulations, and DFT Calculations

**DOI:** 10.1155/jotm/1786204

**Published:** 2025-07-01

**Authors:** Mahmoud A. A. Ibrahim, Alaa M. A. Hassan, Alaa H. M. Abdelrahman, Gamal A. H. Mekhemer, Peter A. Sidhom, Shaban R. M. Sayed, Ashraf M. M. Abdelbacki, Mohamed-Elamir F. Hegazy

**Affiliations:** ^1^Computational Chemistry Laboratory, Chemistry Department, Faculty of Science, Minia University, Minya 61519, Egypt; ^2^School of Health Sciences, University of KwaZulu-Natal, Westville Campus, Durban 4000, South Africa; ^3^Department of Engineering, College of Engineering and Technology, University of Technology and Applied Sciences, Nizwa 611, Oman; ^4^Department of Pharmaceutical Chemistry, Faculty of Pharmacy, Tanta University, Tanta 31527, Egypt; ^5^Department of Botany and Microbiology, College of Science, King Saud University, P.O. Box 2455, Riyadh 11451, Saudi Arabia; ^6^Deanship of Skills Development, King Saud University, P.O. Box 2455, Riyadh 11451, Saudi Arabia; ^7^Department of Pharmaceutical Biology, Institute of Pharmaceutical and Biomedical Sciences, Johannes Gutenberg University, Staudinger Weg 5, Mainz 55128, Germany

**Keywords:** DFT calculations, EBNA1, EBV infection, molecular dynamics simulations, NPACT database

## Abstract

Epstein–Barr nuclear antigen 1 (EBNA1) is an attractive therapeutic target for identifying pharmaceutical drug molecules to fight Epstein–Barr virus (EBV) contagion because of its key function in viral reproduction. To find potent EBNA1 inhibitors, the Naturally Occurring Plant-based Anticancer Compound-Activity-Target (NPACT) database, including > 1500 compounds, was filtered utilizing computational approaches. The efficiency of the docking technique used to anticipate the inhibitor–EBNA1 binding pose was initially evaluated based on obtainable experimental data. Upon the computed docking scores, molecular dynamics simulations (MDSs) were executed for the most superior NPACT compounds bound to EBNA1, accompanied by binding affinity estimations utilizing the MM/GBSA approach. According to binding affinity computations over 200 ns MDS, bitucarpin A demonstrated stronger Δ*G*_binding_ than KWG, an EBNA1 reference inhibitor, with values of −39.1 and −32.4 kcal/mol, respectively. Post-MD analyses assured the steadiness of bitucarpin A inside the EBNA1 binding pocket over 200 ns MDS. Besides, pharmacokinetics, physicochemical, and toxicity features were predicted for bitucarpin A and demonstrated its promising oral bioavailability. Density functional theory calculations were executed, and their outcomes substantiated the results given by docking and MDS computations. According to these findings, bitucarpin A showed promising inhibitory activity as a potent EBNA1 inhibitor that may be a prospective anti-EBV drug candidate.

## 1. Introduction

Epstein–Barr virus (EBV) is a member of the *γ*-herpesvirus family and is an enwrapped virus comprising a linear double-stranded DNA genome [1]. EBV is widely distributed, affecting over 90% of adult humans worldwide, and is primarily transmitted orally [2, 3]. EBV infection is intimately linked to specific human cancers, like NK/T-cell lymphoma and breast carcinoma [4, 5].

During the duration of an infection, EBV exhibits two alternating phases: latent and lytic [6]. New virions are created and discharged from the host cell to infect new target cells during the lytic phase. Both host and viral variables have an influential role in EBV lytic reactivation. The molecular mechanism of the organization of EBV lytic reactivation is still unclear [7].

Recent research indicates that the development of EBV-associated cancers may be attributed to both latent and lytic EBV infections [8]. When the EBV genome is in its dimeric state, the Epstein–Barr nuclear antigen 1 (EBNA1), the only viral protein produced in all EBV-linked malignancies, is essential for transcription, repetition, and genome maintenance [9]. EBNA1 has a strong correlation between latent EBV infection and many epithelial and lymphoid cancers [10–13]. As a result, EBNA1 may be considered a viable and promising target for the treatment of malignancies infected with EBV.

Lately, several peptides and small compounds as EBNA1 inhibitors have been suggested and evolved as anticarcinoma medications [14–16]. Nonetheless, their lack of stability and low emission quantum yield have hindered their continued use in many experimental investigations involving the long-term emission estimation of the EBNA1 localization. Some research has been performed on identifying naturally occurring substances as EBNA1 inhibitors. The hydrophobic nature of natural products (NPs) can be used to improve EBNA1 inhibitory activities and fight EBV [16–18].

Historically, NPs are a source of prospective medications, especially for infectious and cancerous illnesses [19, 20]. The Naturally Occurring Plant-based Anticancer Compound-Activity-Target (NPACT) database primarily concerns naturally occurring anticancerous compounds sourced from plants [21]. NPACT is unprecedented in providing the bioactivities of these natural compounds against different cancer cell lines.  Figure S1 shows that the NPACT database consists of 19 groups; the bulk of NPACT chemicals are terpenoids (33.0%), pursued by flavonoids (21.0%), alkaloids (7.0%), lignans (6.0%), polyketides (6.0%), and simple aromatic NPs (5.0%).

**Figure 1:**
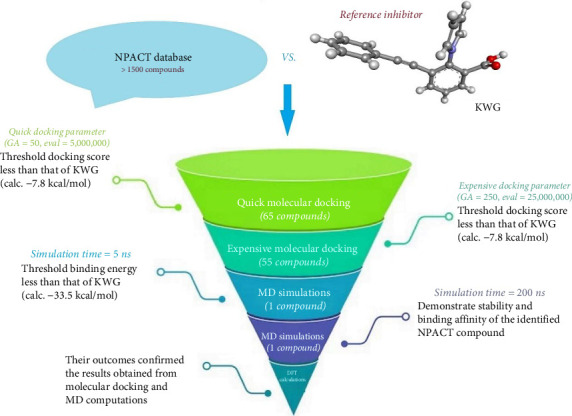
A schematic diagram of the utilized computational techniques and virtual screening of the NPACT database.

Herein, a continuous effort was specialized to hunt plant-derived inhibitors against EBNA1. The NPACT database, containing > 1500 molecules, was mined to identify putative EBNA1 inhibitors using computational approaches. The NPACT database was virtually screened toward EBNA1 using a molecular docking technique. Molecular dynamics simulations (MDSs) were accomplished for the top-ranking NPACT compounds obtained from docking computations, followed by binding affinity assessment utilizing the molecular mechanics/generalized Born surface area (MM/GBSA) approach. The structural and energetical investigations of the best NPACT compound were then scrutinized throughout 200 ns MDS. The oral bioavailability of the identified NPACT compound was determined based on drug-likeness and ADMET features. Moreover, the electrochemical characteristics of the identified NPACT compound were analyzed utilizing DFT computations.  Figure 1 represents a diagram of the utilized computational techniques and virtual screening of the NPACT database. The current work highlights the potentiality of the identified NPACT compound as a promising inhibitor of EBNA1 and a putative drug candidate to combat EBV contagion. The main limitation of this research is the lack of experimental validation for the identified EBNA1 inhibitors, which necessitates further in vitro and in vivo investigations.

## 2. Computational Methodology

### 2.1. Preparation of EBNA1

The human EBNA1 crystal structure (PDB code: 6npp, resolution = 1.35 Å) [22] was retrieved and prepared. For the preparation purpose of ENBA1, water compounds, cocrystal inhibitor, and nonstandard residues were eliminated. Besides, the protonation states were determined for the titratable residues utilizing the H++ webserver [23]. The missing H-atoms were then inserted.

### 2.2. Preparation of NPACT Database

All chemical structures of the NPACT database were initially downloaded and saved in SDF format for database preparation before docking computations [21]. International Chemical Identifier (InChIKey) was utilized to remove the duplicated compounds [24]. The 2D chemical structures of NPACT compounds were then converted into 3D conformers utilizing Omega2 software with a maximum of 200 conformers generated within a 10 kcal/mol energy window [25, 26]. Using SZYBKI software, the generated 3D coordinates were then energetically minimized by the MMFF94S force field [27, 28]. The ionization states of the NPACT compounds were adjusted by the Fixpka function of QUACPAC software [29]. The Gasteiger–Marsili method was employed to compute the atomic charge for NPACT compounds [30]. The prepared NPACT files are accessible on https://www.compchem.net/ccdb.

### 2.3. Docking Computations

All docking computations were performed utilizing AutoDock4.2.6 software [31]. AutoDock Tools was employed to convert PDB into PDBQT format in accordance with the docking protocol [32]. In the present work, two levels of docking computations were carried out: quick and expensive docking estimations. The genetic algorithm (*GA*) was adapted to 50 for quick and 250 for expensive docking estimations, with 5 × 10^6^ and 25 × 10^6^ energy evaluations for quick and expensive docking estimations, respectively. The other docking settings were left at their default values. The dimensions of the grid box were 50 × 50 × 50 Å^3^ along the *x*, *y*, and *z* axes. The grid central coordinates were accurately determined at *X* = −8.253, *Y* = −8.253, and *Z* = −15.597. A grid spacing value of 0.375 Å was utilized in all docking computations.

### 2.4. MDSs

MDS was employed to examine the molecular properties of proteins and ligands and their interactions, solvation, and conformational changes under different circumstances [33, 34]. In the current study, AMBER20 software was utilized to execute the MDS for the investigated NPACT compounds complexed with EBNA1 [35]. More specifics of the utilized protocol for executing MDS are provided elsewhere [36–39]. Concisely, AMBER force field 14SB [40] and general AMBER force field (GAFF2) [41] were utilized to describe the EBNA1 and NPACT compounds, respectively. Utilizing Gaussian09 software, the geometrical optimization for the NPACT compounds was executed at the HF/6-31G∗ level of theory [42]. The atomic charges of the optimized NPACT compounds were evaluated utilizing the restrained electrostatic potential (RESP) approach [43]. The EBNA1–NPACT complexes were then immersed in a 12 Å truncated octahedral box of TIP3P molecules [44]. To neutralize EBNA1–NPACT complexes, chloride or sodium counterions were inserted via tleap implemented inside AMBER20 software. The comprehensive complex was initially energetically minimized for 5000 iterations. Under the NVT ensemble, the minimized systems were warmed up for 50 ps up to 310 K. Under the NPT ensemble, the equilibration stage for the inspected complexes was then realized for 10 ns. Following the equilibration stage, the production phases were run for 5 and 200 ns. Frames were collected every 10 ps along MDS, giving 500 and 20,000 snapshots for 5 and 200 ns MDSs, respectively. The cutoff value was subsequently applied to the nonbonded interactions, utilizing a radius of 12 Å. Frame analyses were performed utilizing the CPPTRAJ module in AMBER20 software. BIOVIA Discovery Studio Visualizer was utilized to generate all graphics in this work [45].

### 2.5. Binding Energy Estimation

According to the single-trajectory approach, the MM/GBSA approach was utilized to estimate the binding energy (Δ*G*_binding_) between the NPACT compounds and EBNA1 over the collected snapshots [46]. The Δ*G*_binding_ was computed as given in Equation (1):

(1) $$\begin{array}{c} {\Delta G}_{\text{binding}}=G_{\text{NPACT}-\text{EBNA}1}-G_{\text{NPACT}}{-G}_{\text{EBNA}1}, \end{array}$$

where the energy term (*G*) is expressed as follows:

(2) $$\begin{array}{c} G=E_{\text{vdW}}+G_{\text{SA}}+E_{\text{ele}}+G_{\text{GB}}-T\Delta S. \end{array}$$

The *E*_vdW_ represents van der Waals energy. *E*_ele_ points out electrostatic energy. *G*_GB_ and *G*_SA_ refer to polar and nonpolar solvation-free energy participation, respectively. Utilizing the modified GB model (igb = 2, developed by Onufriev and colleagues), the *G*_GB_ was calculated [47]. *T* and *S* stand for the absolute temperature and entropy, respectively. However, the entropy contribution was overlooked in the current study due to its high computational costs.

### 2.6. Drug-Likeness Characteristics

SwissADME webserver was applied to predict the drug-likeness features of the identified NPACT compound [48]. Two rules, namely Veber's rule and Lipinski's rule of five [49], were used for inspecting drug-likeness characteristics. Lipinski's rule of five recommends that the number of hydrogen bond donors and acceptors (nHBD and nHBA, respectively) should be lower than or equal to 5 and 10, respectively. Besides, molecular weight (MW) and octanol–water partition coefficient (Mlog *p*) should be lower than or equal to 500 Da and 5, respectively. However, Veber's rule is more concerned with topological polar surface area (TPSA ≤ 140 Å^2^) and the number of rotatable bonds (*n*_rot_ ≤ 10).

### 2.7. Pharmacokinetics and Toxicity Features

Absorption, distribution, metabolism, excretion, and toxicity (ADMET) characteristics are essential in remedy and chemical peril evaluations. Consequently, the pharmacokinetic and toxicity characteristics were anticipated using the pkCSM server [50]. The absorption of the investigated NPACT compound was determined according to human intestinal absorption (HIA). Based on the volume of distribution (VDss), the distribution of the investigated compound was evaluated. The metabolism and excretion properties were estimated by cytochrome P450 inhibitors/substrates and renal OCT2 substrate, respectively. The toxicity was estimated on the basis of AMES toxicity.

### 2.8. DFT Computations

All DFT computations were performed utilizing Gaussian09 software [42]. For DFT calculations, the final snapshot was obtained from MDS over 200 ns. The investigated systems were initially optimized at the M062X/6-311+G^∗∗^ level of theory. To point out the nucleophilic and electrophilic regions, molecular electrostatic potential (MEP) maps were created for the optimized systems utilizing a 0.002 a.u electron density envelope [51, 52]. The frontier molecular orbital (FMO) theory was also applied in order to provide a sufficient illustration of the electronic properties of the inspected systems [53]. In this regard, the highest occupied and lowest unoccupied molecular orbitals (HOMO and LUMO, respectively) were graphed for the inspected systems. In the same manner, energies of LUMO and HOMO (i.e., *E*_LUMO_ and *E*_LUMO_, respectively) were estimated. The *E*_gap_ value was then computed as demonstrated in equation (3).

(3) $$\begin{array}{c} E_{\text{gap}}=E_{\text{LUMO}}-E_{\text{HOMO}}. \end{array}$$

The quantum mechanical descriptors, namely, ionization potential (*IP*), electron affinity (*EA*), global hardness (*η*), and global softness (*S*), were estimated for the inspected systems, as illustrated by equations (4)–(7).

(4) $$\begin{array}{c} IP=-E_{\text{HOMO}}, \end{array}$$

(5) $$\begin{array}{c} EA=-E_{\text{LUMO}}, \end{array}$$

(6) $$\begin{array}{c} \eta =\frac{E_{\text{LUMO}}-E_{\text{HOMO}}}{2}, \end{array}$$

(7) $$\begin{array}{c} S=\frac{1}{\eta }. \end{array}$$

## 3. Results and Discussion

### 3.1. Docking Validation

**Figure 2:**
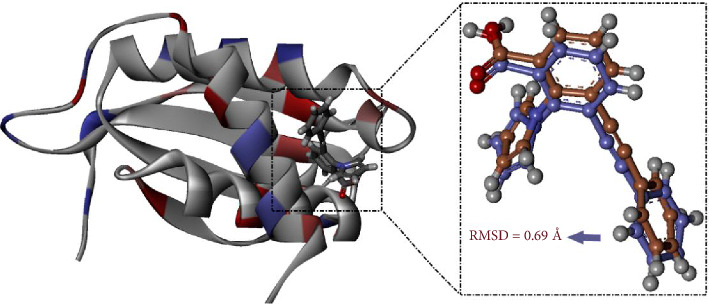
Superposition of the native binding pose (mauve) and the foretold docking mode (orange) of KWG toward EBNA1.

Before proceeding with the virtual screening of the NPACT database, the utilized docking protocol was initially validated. For docking validation purposes, the cocrystal KWG ligand was extracted from its X-ray crystal structure and redocked toward EBNA1. The foretold docking mode was compared with its native binding pose, and the corresponding root-mean-square deviation (RMSD) was estimated. The computed RMSD was found to be 0.69 Å (Figure 2) [54–56]. Such a promising RMSD value of < 2.0 Å implied the validity of the used docking technique and its applicability for permissible prediction of the correct binding mode of NPACT compounds against EBNA1.

In the realm of medicinal chemistry, the high electron density of the pyrrole ring present in KWG (VK-0941 or 3-(phenylethynyl)-2-(1H-pyrrol-1-yl)benzoic acid) is responsible for its molecular interactions within the EBNA1 binding pocket. KWG demonstrated a promising IC_50_ with a value of 0.89 μM in comparison with two compounds, namely VK-0064 (2-pyrrolo-benzoic acid) and VK-0044 (5-phenylacetylenyl nicotinic acid), which exhibited IC_50_ values of 2.0 and 110 μM, respectively [22]. As well, the crystal structure of KWG in complex with EBNA1 is available [22]. For the aforementioned reasons, KWG was employed as the positive control in this investigation.

### 3.2. Screening of NPACT Database

Virtual screening has grown in popularity and effectiveness because of its potential to quickly filtrate through millions of small compounds and lessen the time and expense associated with the drug discovery process [57]. Herein, the NPACT database was mined against EBNA1 using quick and expensive computations (see Section 2.3 for more details). At the outset, quick docking computations were executed for the whole NPACT database toward EBNA1 with *GA* = 50 and *eval* = 5 × 10^6^. The quick binding scores were predicted and are gathered in Table S1. As evident in Table S1, only 65 NPACT compounds revealed binding scores lower than KWG (calc. −7.8 kcal/mol). As a result, these 65 NPACT compounds underwent expensive docking computations with *GA* = 250 and *eval* = 25 × 10^6^, and the predicted binding scores are enrolled in Table S2. Interestingly, 55 out of 65 NPACT compounds demonstrated less binding scores than KWG (calc. −7.8 kcal/mol). The estimated binding scores of these 55 NPACT compounds are summarized in Table 1, and their binding modes with EBNA1 are given in Figure S2. As displayed in Figure S2, most of the identified NPACT compounds demonstrated approximately identical binding modes within the binding pocket of EBNA1, exhibiting H-bonds with LYS586, LYS477, and ASN519 inside the binding pocket of EBNA1.

**Figure 3:**
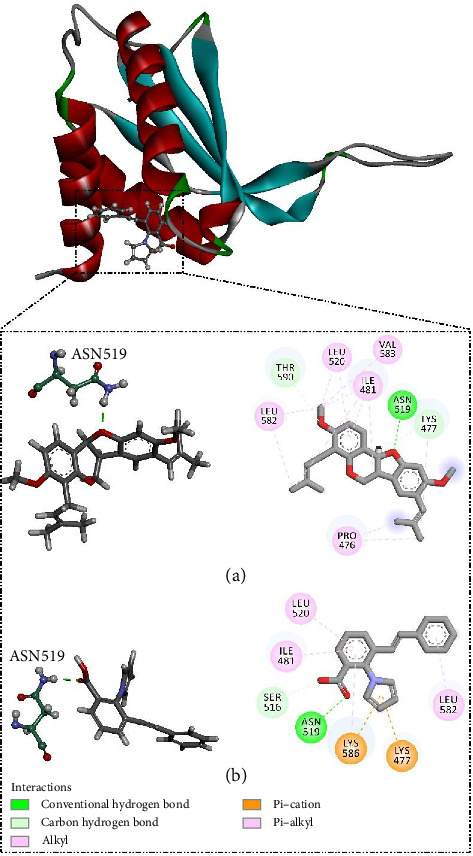
3D and 2D EBNA1–inhibitor interactions of the predicted docking poses of (a) bitucarpin A (NPACT01468) and (b) KWG toward EBNA1.

Bitucarpin A (NPACT01468), an NP separated from *Bituminaria bituminosa*, unveiled the lowest binding score toward EBNA1 (calc. −9.7 kcal/mol). The docking mode of bitucarpin A inside the binding pocket of EBNA1 was similar to the cocrystal KWG ligand. More precisely, the tetrahydrofuran ring of bitucarpin A formed an influential H-bond with NH_2_ of ASN519 (2.0 Å) (Figure 3). Besides, bitucarpin A demonstrated two carbon H-bonds with LYS477 and THR580. Bitucarpin A also demonstrated alkyl and pi–alkyl interactions with PRO476, ILE481, LEU520, LEU582, and VAL583 (Figure 3).

In comparison with bitucarpin A, the cocrystal KWG ligand unveiled a higher binding score of −7.8 kcal/mol toward EBNA1. Inspecting its binding mode, the carboxylic acid of KWG exhibited only an H-bond with the NH_2_ of ASN519 (2.09 Å) (Figure 3). KWG also exhibited pi–cation interactions with LYS477 and LYS586.

### 3.3. MDSs

MDS is usually utilized to assess the docking outcomes and gain deeper insight into the steadiness of the inhibitor–receptor complex [33, 34]. Moreover, MDS is employed to demonstrate the characteristics, pattern, and strength of inhibitor–receptor interactions in addition to conformational variations of the inspected complexes. The most promising 55 NPACT compounds bound to EBNA1 were subjected to 5 ns MDS to lessen the computational time and cost. The corresponding Δ*G*_binding_ was evaluated utilizing the MM/GBSA approach (Table S3). From the summarized data in Table S3, only bitucarpin A (NPACT01468) in complex with EBNA1 manifested lower binding energy with a Δ*G*_binding_ value of −38.0 kcal/mol, in comparison with KWG (Δ*G*_binding_ = −33.5 kcal/mol). Of note, both docking scores and MM/GBSA energies serve as relative binding energies, illustrating the potency ranking of inhibitors bound to a protein. Nonetheless, the binding energies calculated via the MM/GBSA approach, which relies on MDS, are deemed more dependable than conventional docking scores, as the former accounts for the flexibility of both the inhibitor and the protein. In order to get more trustworthy outcomes, MDS for bitucarpin A in complex with EBNA1 was elongated to 200 ns accompanied by binding affinity evaluations (Table 2). Notably, there was no discernible change between the computed Δ*G*_binding_ for bitucarpin A in complex with EBNA1 over the 5 and 200 ns MDS. Several prior investigations have indicated that MDS of EBNA1 complexed with various inhibitors, conducted over a simulation duration of 100–200 ns, are adequate for the computation of binding energies and subsequent postsimulation analyses [58, 59]. Upon the data listed in Table 2, bitucarpin A disclosed greater binding affinity in comparison with KWG complexed with EBNA1 over a 200 ns MDS with average Δ*G*_binding_ values of −39.1 and −32.4 kcal/mol, respectively. These results outlined that bitucarpin A and KWG are promising EBNA1 inhibitors and may be probable anti-EBV drug candidates. The main limitation of the current research is the absence of experimental validation for the identified EBNA1 inhibitors, necessitating additional in vitro and in vivo assessments.

**Figure 4:**
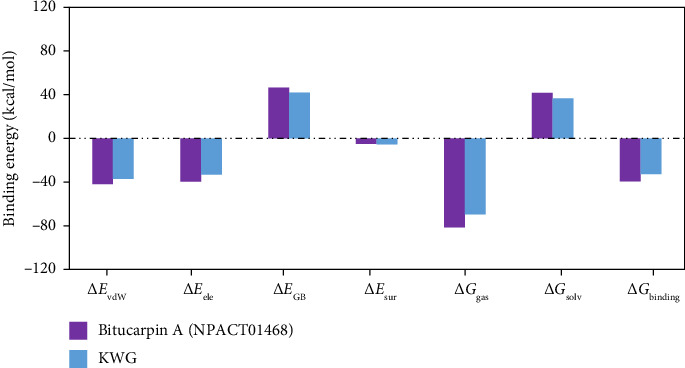
Binding energy components of bitucarpin A (NPACT01468)- and KWG–EBNA1 complexes over 200 ns MDS.

To gain detailed insights into the nature of the main interactions in receptor–inhibitor binding, the MM/GBSA binding energy was decomposed into its individual components (Figure 4). As illustrated in Figure 4, the binding affinity of bitucarpin A and KWG bound to EBNA1 was dominated by *E*_vdW_ with average values of −41.8 and −36.7 kcal/mol, respectively. Additionally, *E*_ele_ was a favorable contributor for bitucarpin A–EBNA1 and KWG–EBNA1 binding energies with average values of −39.2 and −32.6 kcal/mol, respectively.

**Figure 5:**
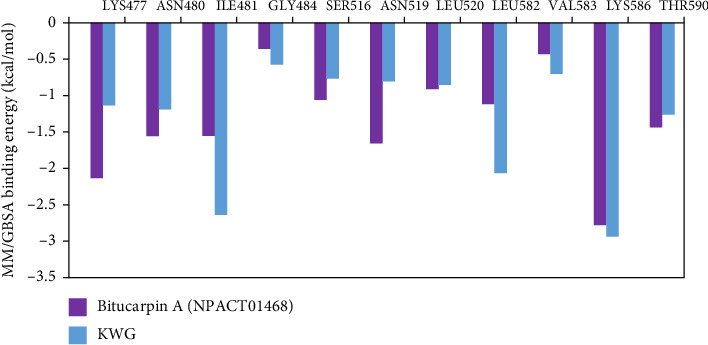
Per-residue energy decomposition analysis for bitucarpin A (NPACT01468)- and KWG–EBNA1 complexes throughout 200 ns MDS.

To investigate the participation of the proximal residues to the inhibitor–EBNA1 binding affinity, the Δ*G*_binding_ was decomposed per residue. Only amino acids with Δ*G*_binding_ < −0.5 kcal/mol were taken into account and are depicted in Figure 5. From Figure 5, LYS586 showed a major favorable contribution to Δ*G*_binding_ with values of −2.7 and −2.9 kcal/mol for bitucarpin A–EBNA1 and KWG–EBNA1 complexes, respectively. Additionally, per-residue decomposition analysis also unveiled an intrinsic contribution of ILE481, LYS477, LEU582, and ASN519 in the binding of bitucarpin A and KWG with EBNA1.

### 3.4. Post-MD Analyses

To assess the steadiness of bitucarpin A–EBNA1 and KWG–EBNA1 complexes over 200 ns MDS, structural and energetical analyses were executed. Four parameters were estimated, including binding energy per frame, RMSD, center-of-mass (CoM) distance, and radius of gyration (Rg).

#### 3.4.1. RMSD Analysis

**Figure 6:**
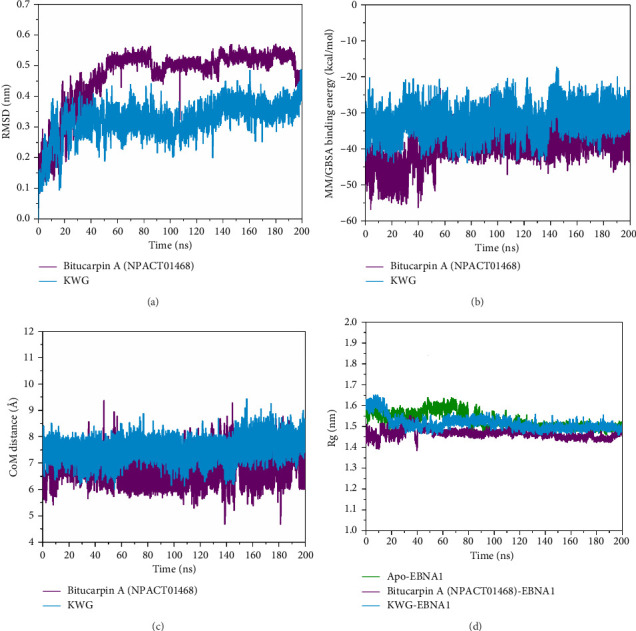
(a) RMSD of C_*α*_ with respect to their respective initial structures, (b) binding energy per frame, (c) CoM, and (d) Rg of apo–EBNA1 (green), bitucarpin A (NPACT01468)–EBNA1 (purple), and KWG–EBNA1 (light blue) over 200 ns MDS.

To obtain deeper insights into the conformational changes of bitucarpin A–EBNA1 and KWG–EBNA1 complexes, the C_*α*_ RMSD fluctuation of the comprehensive complex was measured over 200 ns MDS (Figure 6(a)). According to Figure 6(a), the investigated complexes reached their dynamic equilibration at the first 10 ns of the MDS, suggesting adequate stability. The average RMSD values were 0.47 and 0.32 nm for bitucarpin A–EBNA1 and KWG–EBNA1 complexes, respectively. Generally, these findings revealed that bitucarpin A and KWG are tightly bound to EBNA1 and do not impact the EBNA1 structural steadiness over 200 ns MDS.

#### 3.4.2. Binding Energy Per Frame

To estimate the thorough energetical steadiness of bitucarpin A–EBNA1 and KWG–EBNA1 complexes over the 200 ns MDS, the correlation between the binding energy and time was plotted in Figure 6(b). From Figure 6(b), the comprehensive steady for bitucarpin A–EBNA1 and KWG–EBNA1 complexes was noticed with Δ*G*_binding_ values of −39.1 and −32.4 kcal/mol, respectively. The most evident result of this analysis is that all complexes remained stable over 200 ns MDS.

#### 3.4.3. CoM Distance

The CoM distance between the identified NPACT compound/KWG and ASN519 was estimated to obtain a deeper comprehension of the structural steadiness of the inspected compounds within the EBNA1 binding pocket over 200 ns MDS (Figure 6(c) and Figure S3). As displayed in Figure 6(c), the evaluated CoM distances were stable for bitucarpin A–EBNA1 and KWG–EBNA1 complexes with mean values of 6.9 and 7.5 Å, respectively. The most apparent result from the CoM distance is that the investigated compounds were bound tightly to EBNA1.

#### 3.4.4. Rg Analysis

To inspect the compactness of the inhibitor–ENBA1 complex, the correlation between Rg and time was evaluated over the 200 ns MDS (Figure 6(d)). Lower Rg values imply a great steadiness and compact structure and vice versa. The average Rg values for apo–EBNA1, bitucarpin A–EBNA1, and KWG–EBNA1 were 1.53, 1.46, and 1.51 nm, respectively (Figure 6(d)). These results demonstrated that the binding of bitucarpin A and KWG significantly stabilized the EBNA1 structure.

### 3.5. Drug-Likeness Properties

The drug-likeness features of bitucarpin A and KWG were computed by the SwissADME webserver and are outlined in Table 3. As listed in Table 3, the investigated compounds obey Lipinski's rule of five without any violation. The MWs of the bitucarpin A and KWG were 352.4 and 287.3 Da, respectively. Besides, the nHBD of the bitucarpin A and KWG were 0 and 1, respectively, while bitucarpin A and KWG demonstrated good nHBA with values of 4 and 2, respectively. The Mlog* P* of bitucarpin A and KWG was promising, with values of 3.2 and 3.4, respectively. The TPSA values of bitucarpin A and KWG were 36.9 and 85.2 Å^2^, respectively, indicating that these inspected compounds had good membrane permeability. Besides, the *n*_rot_ of the bitucarpin A and KWG were 5 and 2, respectively. Notably, the physicochemical features of the investigated compounds demonstrated their oral bioavailability.

**Figure 7:**
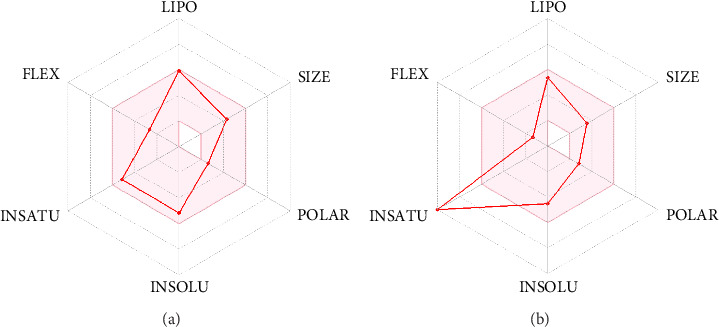
The bioavailability radar plots of (a) bitucarpin A (NPACT01468) and (b) KWG.

Figure 7 represents the bioavailability radar plot created by the SwissADME webserver. The radar plot displays important physicochemical characteristics like hydrophobicity (LIPO), insolubility (INSOLU), unsaturation (INSATU), rotatable bonds (FLEXI), MW (SIZE), and polar surface area (POLAR). The LIPO (XLOGP3) should range from −0.7 to +5.0. SIZE, POLAR, and FLEXI should be < 500 g/mol, < 140 Å^2^, and < 10, respectively. INSATU should be < 6. INSATU should lie between 0.25 and 1.0. The bitucarpin A and KWG radar plots revealed that the investigated compounds had favorable physicochemical characteristics.

### 3.6. Pharmacokinetic and Toxicity Properties

Prediction of ADMET characteristics plays a critical role in the drug design process [60]. Consequently, the ADMET characteristics of bitucarpin A and KWG were predicted utilizing the pKCSM webserver and are compiled in Table 4. From Table 4, bitucarpin A and KWG demonstrated promising HIA with values of 98.3% and 96.6%, respectively, implying these compounds can be facilely absorbed by the intestine. The predicted log VDss were 0.316 and −0.332 L/kg for bitucarpin A and KWG, respectively, indicating that these compounds are distributed in tissues rather than plasma. Additionally, metabolizing enzymes are very important in the phase I drug design process. Bitucarpin A and KWG are found to be substrates and inhibitors for CYP3A4 and CYP1A2, respectively (Table 4). Besides, bitucarpin A is an inhibitor for CYP2C19 and CYP2C9. However, KWG is a noninhibitor for CYP2C19 and CYP2C9. For excretion property, bitucarpin A and KWG are nonsubstrates for renal OCT2. For inspecting the toxicity property, the AMES test outcomes suggested that bitucarpin A and KWG are inactive as AMES toxicity.

### 3.7. DFT Calculations

**Figure 8:**
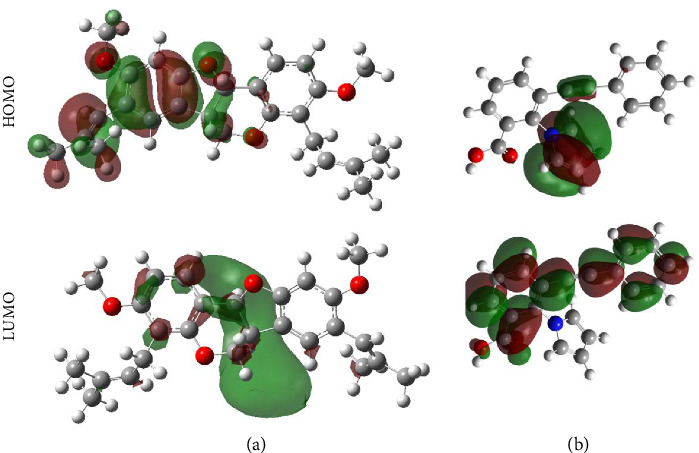
Plots of HOMO and LUMO distribution patterns of (a) bitucarpin A (NPACT01468) and (b) KWG.

Within the frame of FMO theory, the electronic parameters for bitucarpin A and KWG were determined. In this regard, *E*_LUMO_, *E*_HOMO,_ and *E*_gap_ were computed to reveal the ability of the investigated compounds to accept and donate electrons (Table 5).  Figure 8 demonstrates the molecular orbital distribution patterns of bitucarpin A and KWG.

As evident in Table 5, the bitucarpin A and KWG demonstrated low *E*_gap_ with values of 6.82 and 6.03 eV, suggesting their high degree of chemical reactivity. As depicted in Figure 8, the HOMO orbitals were fundamentally noticed above the nucleophilic regions (i.e., N and O atoms) of the investigated compounds, while LUMO levels were noticed above the electrophilic sites (i.e., H atoms).

Numerous global reactivity descriptors, involving *IP*, *EA*, *η*, and *S*, of bitucarpin A and KWG were estimated (Table 5). Quantum mechanical descriptors are substantial in examining the steadiness and reactivity of the investigated compounds. As given in Table 5, the *EA* was found with values of −0.11 and 1.14 eV for bitucarpin A and KWG, respectively. Bitucarpin A and KWG demonstrated *IP* with values of 6.71 and 7.17 eV, respectively. Upon the data listed in Table 5, the hardness (*η)* values of bitucarpin A and KWG were 3.41 and 3.01 eV, respectively. Bitucarpin A and KWG exhibited softness (*S*) with values of 0.29 and 0.33 eV, respectively. According to global reactivity descriptors, bitucarpin A demonstrated better chemical stability and reactivity compared to the cocrystallized KWG inhibitor. It is worth noting that these results were in line with the outcomes obtained from docking computations and MDS.

**Figure 9:**
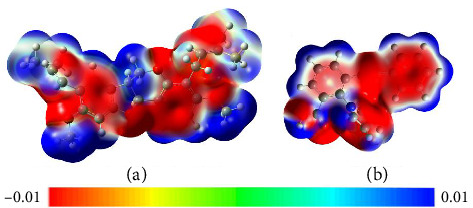
The MEP maps of the final frame of (a) bitucarpin A (NPACT01468) and (b) KWG.

MEP maps of bitucarpin A and KWG are plotted in Figure 9. From Figure 9, the nucleophilic and electrophilic centers were manifestly noticed within the investigated compounds by the existence of red- and blue-colored zones, respectively. Notably, the electronegative sites were positioned over N and O atoms, while electropositive sites were situated around the H atoms. By analyzing MEP maps of the investigated compounds, these compounds demonstrated the capacity to exhibit H-bonds with the fundamental residues inside the EBNA1 binding pocket.

### 3.8. Bitucarpin A Analogs as EBV Drug Candidates

According to the promising affinity of bitucarpin A as an EBNA1 inhibitor, the current study was augmented to inspect the potentiality of the bitucarpin A analogs. PubChem database was explored to hunt bitucarpin A analogs, and the collected compounds were prepared for docking calculations against EBNA1.  Table 6 lists the binding scores and 2D chemical structures of investigated bitucarpin A analogs. It is apparent from Table 6 that the estimated binding scores for bitucarpin A analogs ranged from −6.5 to −9.3 kcal/mol. A comparison of the binding scores demonstrated that bitucarpin A unveiled the lowest binding score (calc. −9.7 kcal/mol) (Table 6).

## 4. Conclusion

EBV is regarded as a significant human pathogen because of its role in infections and cellular cancers. About 90% of the global population is reportedly infected with this oncolytic virus. EBNA1 plays a crucial function in DNA reproduction and transcription starting of viral and cellular genes, and for this reason, it is considered a putative druggable target. Herein, the NPACT database was mined as prospective EBNA1 inhibitors with the assistance of integrated docking computations and MDS, accompanied by binding affinity estimations. Depending on the binding energy computations over 200 ns MDS, bitucarpin A (NPACT01468) demonstrated stronger binding energy with an average Δ*G*_binding_ value of −39.1 kcal/mol, in comparison with KWG (calc. −32.4 kcal/mol). The stability of the bitucarpin A and KWG complexed with EBNA1 was proven utilizing structural and energetical analyses throughout 200 ns MDS. According to drug-likeness and ADMET features, bitucarpin A and KWG demonstrated good oral bioavailability. Additionally, the DFT computations aligned with the outcomes obtained from docking computations and MDS. These findings proposed bitucarpin A as a potential anti-EBV drug candidate and could be promising for additional experimental assays.

## Figures and Tables

**Table 1:** Estimated quick and expensive binding scores of the promising 55 NPACT compounds toward EBNA1.^a^

Compound Name/ID	Docking score (kcal/mol)	
	Quick	Expensive
KWG	−7.8	−7.8
NPACT01468	−8.0	−9.7
NPACT00148	−9.7	−9.5
NPACT00124	−9.3	−9.3
NPACT00774	−8.9	−9.2
NPACT01326	−9.2	−9.1
NPACT01325	−9.0	−9.0
NPACT00309	−9.0	−9.0
NPACT01034	−8.6	−9.0
NPACT01327	−9.0	−8.8
NPACT01270	−8.4	−8.8
NPACT00382	−8.7	−8.7
NPACT01268	−8.6	−8.6
NPACT00307	−8.6	−8.6
NPACT00306	−8.6	−8.5
NPACT00189	−8.4	−8.5
NPACT01496	−8.5	−8.4
NPACT01197	−8.2	−8.4
NPACT01410	−8.4	−8.4
NPACT01273	−8.0	−8.4
NPACT00123	−8.3	−8.4
NPACT01271	−8.2	−8.4
NPACT01342	−8.4	−8.4
NPACT01155	−8.4	−8.4
NPACT01000	−8.2	−8.4
NPACT00305	−8.3	−8.4
NPACT01200	−8.3	−8.4
NPACT00017	−8.3	−8.3
NPACT00829	−8.0	−8.3
NPACT00304	−8.2	−8.3
NPACT00446	−8.2	−8.3
NPACT00062	−8.3	−8.3
NPACT01049	−8.2	−8.3
NPACT01226	−8.2	−8.2
NPACT00475	−8.1	−8.2
NPACT00995	−8.2	−8.2
NPACT00909	−8.0	−8.2
NPACT00700	−8.1	−8.1
NPACT00576	−7.9	−8.1
NPACT00560	−8.1	−8.1
NPACT00195	−8.0	−8.0
NPACT00034	−7.9	−8.0
NPACT01483	−7.9	−7.9
NPACT01248	−7.8	−7.9
NPACT00558	−7.9	−7.9
NPACT01125	−7.9	−7.9
NPACT01427	−7.9	−7.9
NPACT00549	−7.9	−7.9
NPACT01341	−7.9	−7.9
NPACT01209	−7.8	−7.8
NPACT00289	−7.8	−7.8
NPACT00176	−7.8	−7.8
NPACT00075	−7.8	−7.8
NPACT01154	−7.8	−7.8
NPACT00557	−8.1	−7.8
NPACT01329	−7.8	−7.8

^a^Data sorted in accordance with the expensive binding scores.

**Table 2:** Evaluated binding energies for bitucarpin A (NPACT01468) and KWG complexed with EBNA1 over 5 and 200 ns MDS.

Compound Name/ID	MM/GBSA binding energy (kcal/mol)	
	5 ns	200 ns
KWG	−33.5	−32.4
Bitucarpin A (NPACT01468)	−38.0	−39.1

**Table 3:** Physicochemical characteristics of bitucarpin A (NPACT01468) and KWG as potential anti-EBV drug candidates.

Compound name/ID	MW (Da)	nHBD	nHBA	TPSA (Å^2^)	M log *p*	*n*_rot_
KWG	287.3	1	2	85.2	3.4	2
Bitucarpin A (NPACT01468)	352.4	0	4	36.9	3.2	5

**Table 4:** ADMET characteristics for bitucarpin A (NPACT01468) and KWG.

ADMET characteristics	KWG	Bitucarpin A (NPACT01468)
*Absorption (A)*		
HIA	96.6%	98.3%

*Distribution (D)*		
Log VDss	−0.332	0.316

*Metabolism (M)*		
CYP1A2 inhibitor	Yes	Yes
CYP3A4 substrate	Yes	Yes
CYP2C9 inhibitor	No	Yes
CYP2C19 inhibitor	No	Yes

*Excretion (E)*		
Renal OCT2 substrate	No	No

*Toxicity (T)*		
AMES test	No	No

**Table 5:** Estimated quantum mechanical descriptors for the optimized bitucarpin A (NPACT01468) and KWG.

Compound name/code	*E*_HOMO_ (eV)	*E*_LUMO_ (eV)	*E*_gap_ (eV)	*EA* (eV)	*IP* (eV)	*η* (eV)	*S* (eV^−1^)
KWG	−7.17	−1.15	6.03	1.14	7.17	3.01	0.33
Bitucarpin A (NPACT01468)	−6.71	0.11	6.82	−0.11	6.71	3.41	0.29

**Table 6:** Estimated binding scores and 2D chemical structures of bitucarpin A analogs toward EBNA1.

Compound name/PubChem ID	Docking score (kcal/mol)	2D chemical structures
PubChem21576494 (bitucarpin A)	−9.7	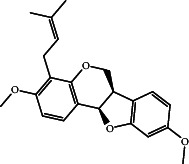
PubChem162999091	−9.3	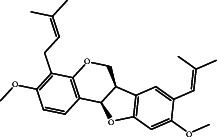
PubChem46883424 (nutiducol)	−8.8	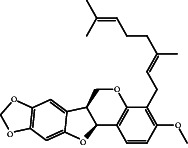
PubChem163020715	−8.4	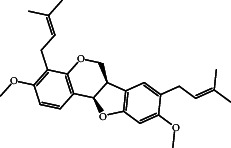
PubChem101633767	−8.0	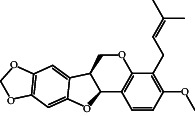
PubChem163064208	−8.0	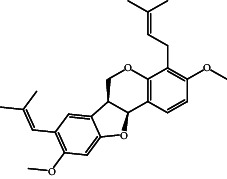
PubChem23242600	−7.9	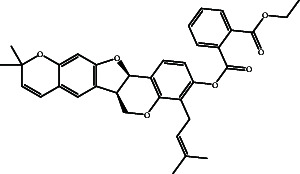
PubChem163020714	−7.9	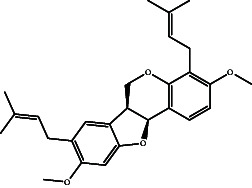
PubChem10076247	−7.6	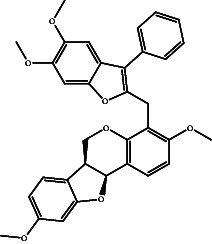
PubChem14357388	−7.4	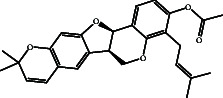
PubChem163064207	−7.3	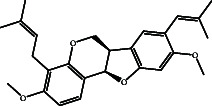
PubChem11079069	−7.1	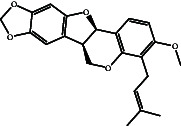
PubChem163020713	−6.8	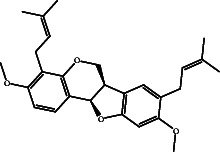
PubChem11566520	−6.5	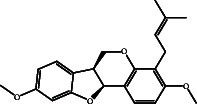

## Data Availability

The data that support the findings of this study are available in the supporting information of this article.

## Supporting Information

Supporting Information: Figure S1. Cancerwise distribution of the compounds in NPACT. Figure S2. 2D representations of the anticipated binding modes for the promising 55 NPACT compounds with the EBNA1 active site. Figure S3. CoM distances of (a) bitucarpin A (NPACT01468) and (b) KWG and ASN519 of the EBNA1 over 200 ns MDS. Table S1. The anticipated quick docking scores for the NPACT compounds and KWG toward EBNA1. Table S2. Estimated quick and expensive binding scores of the promising 65 NPACT compounds toward EBNA1. Table S3. The anticipated quick and expensive binding scores and MM/GBSA binding energies over 5 ns for the top 55 NPACT compounds and KWG toward EBNA1.
