# Supplementary material for: In Silico Mining of NPACT Database Toward Identification of EBNA1 Inhibitor: Virtual Screening, Molecular Dynamics Simulations, and DFT Calculations
Source: J Trop Med. 2025 Jul 1;2025:1786204. doi: 10.1155/jotm/1786204 (PMC12237558; doi:10.1155/jotm/1786204)
Supplement: Supporting Information — Additional supporting information can be found online in the Supporting Information section. [file 1786204.f1.docx]

#
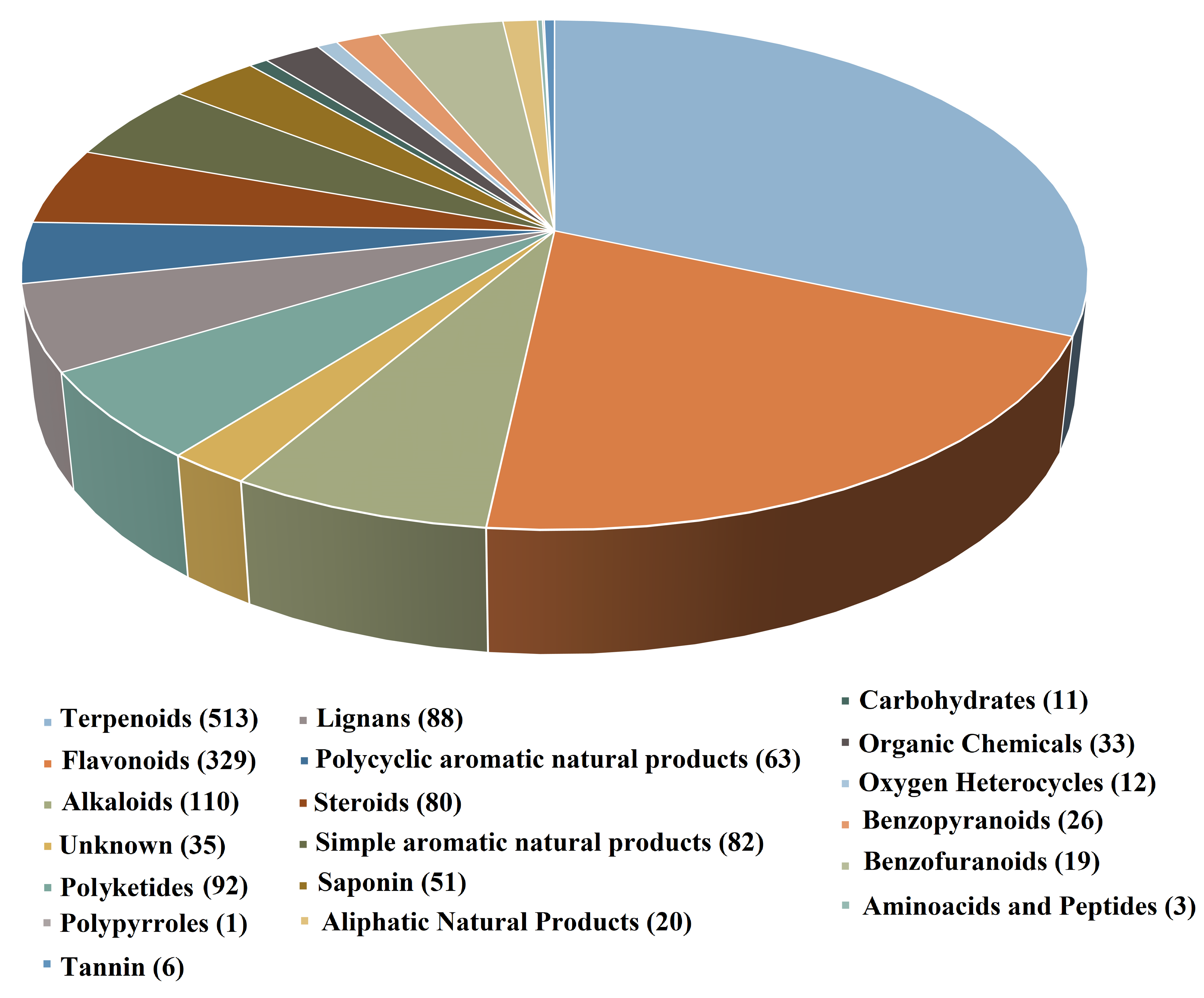


**Figure S1.** Cancer–wise distribution of the compounds in NPACT.





**Figure S2.** 2D representations of the anticipated binding modes for the promising 55 NPACT compounds with the EBNA1 active site.


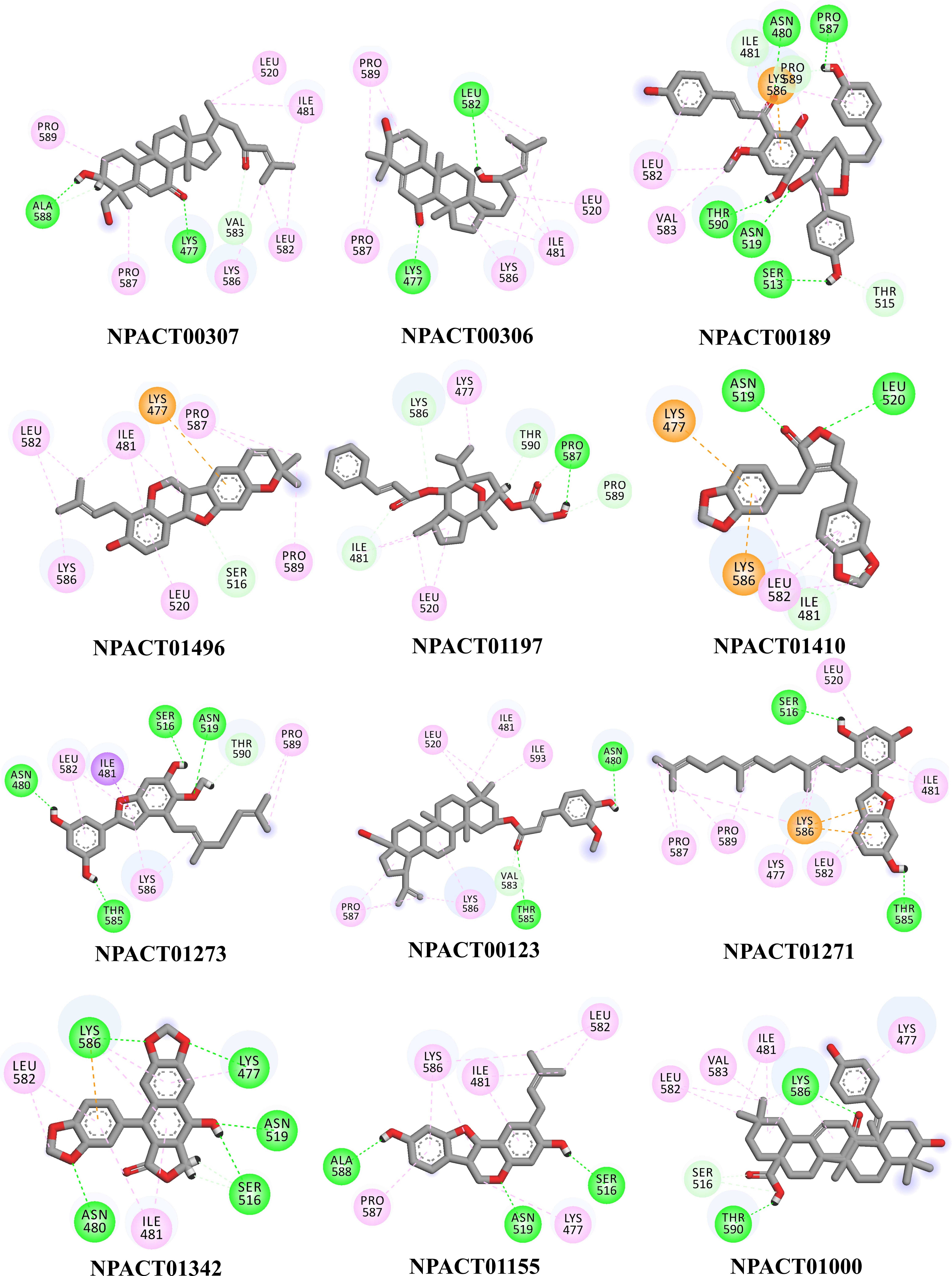


**Figure S2.** *Continued.*


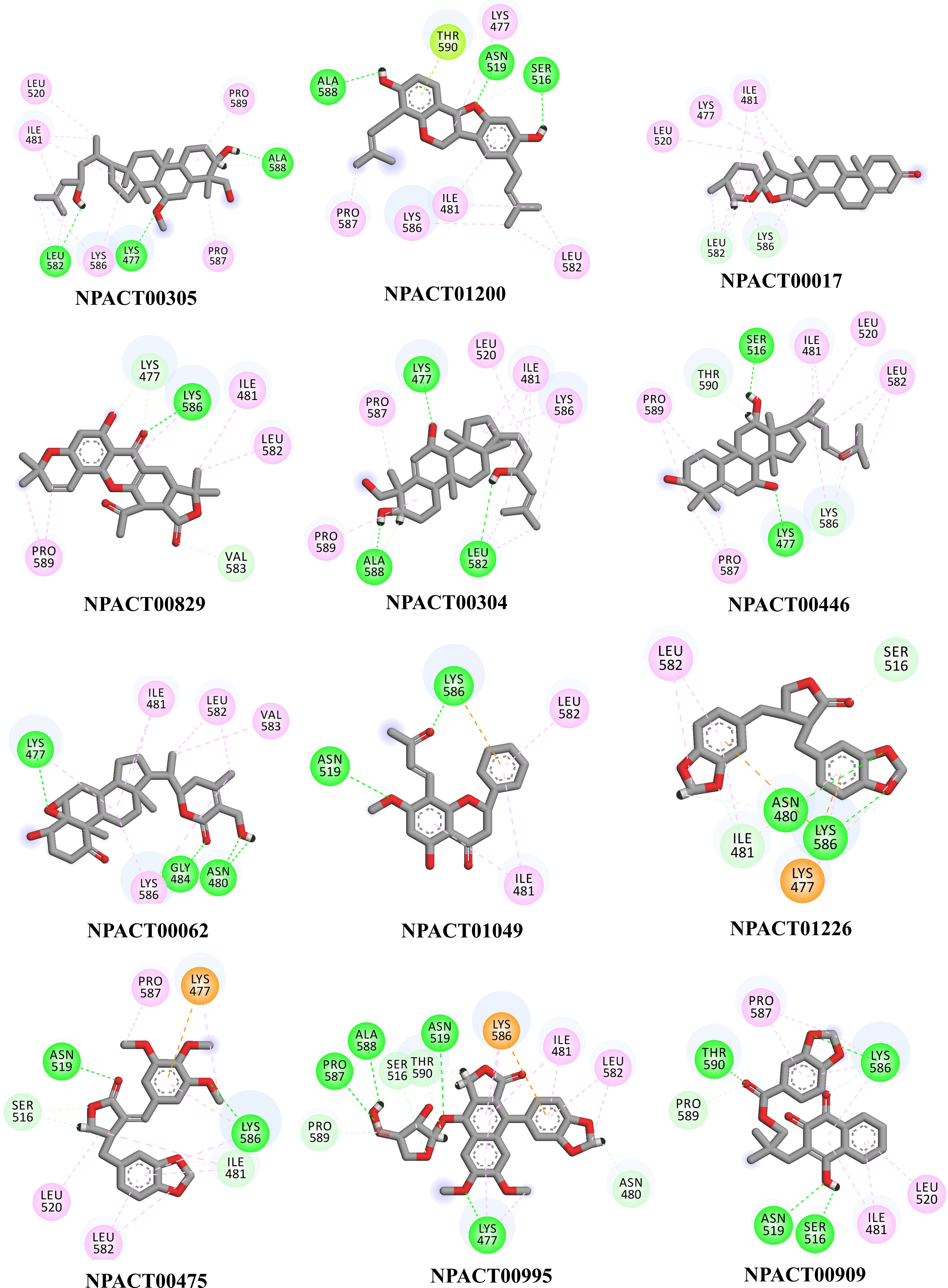


**Figure S2.** *Continued.*

*
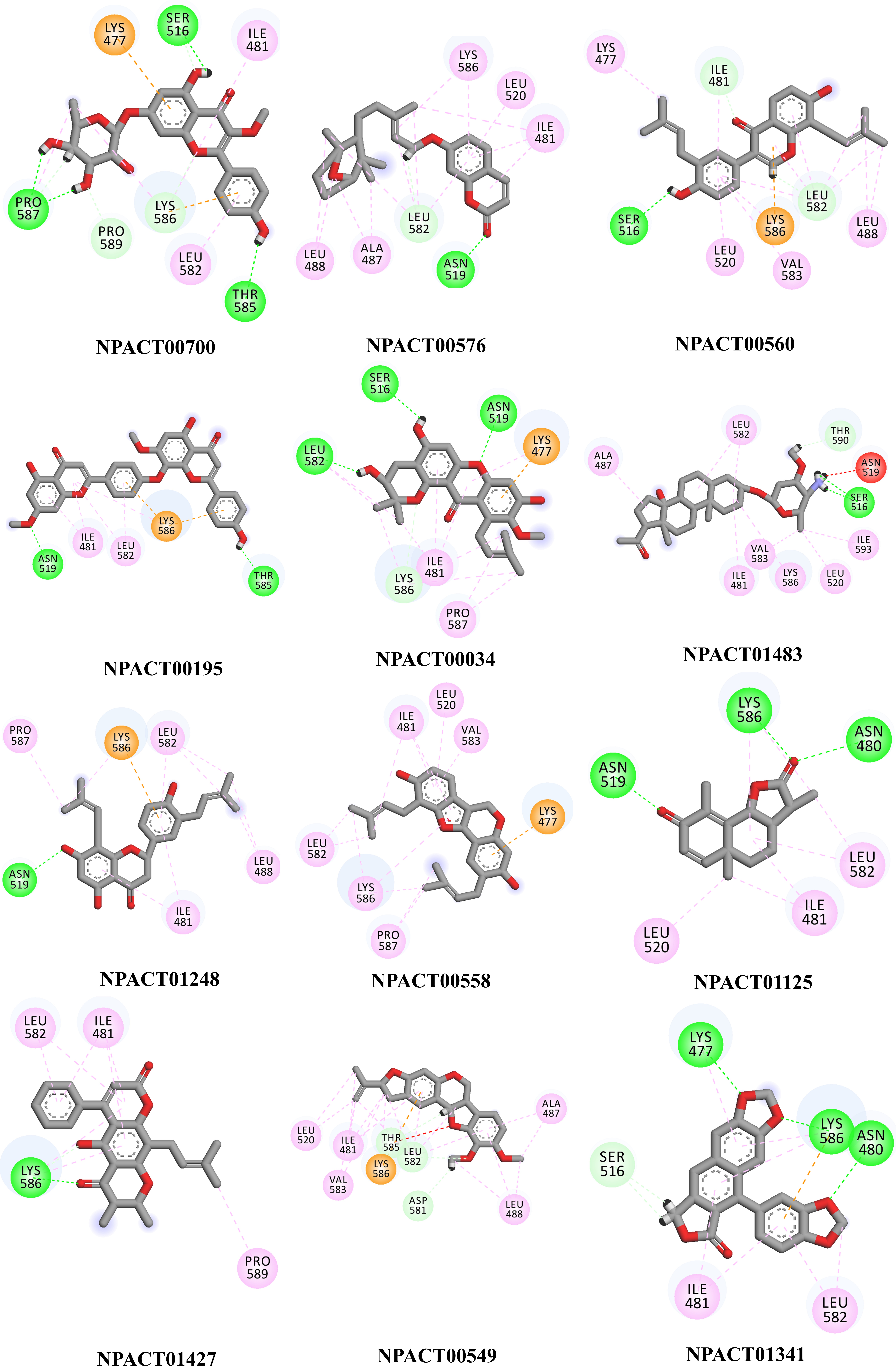
*

**Figure S2.** *Continued.*

*
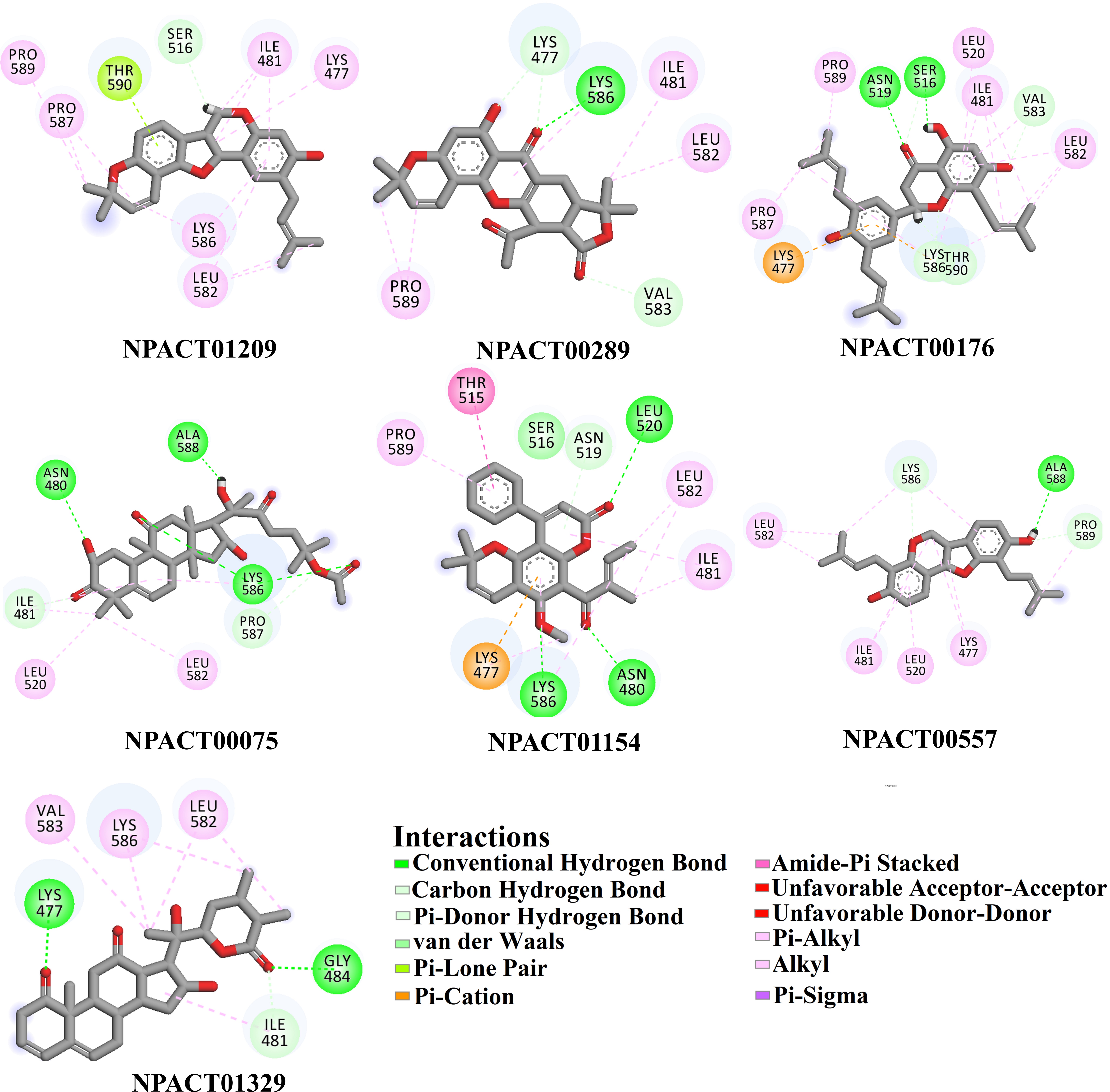
*

**Figure S2.** *Continued.*


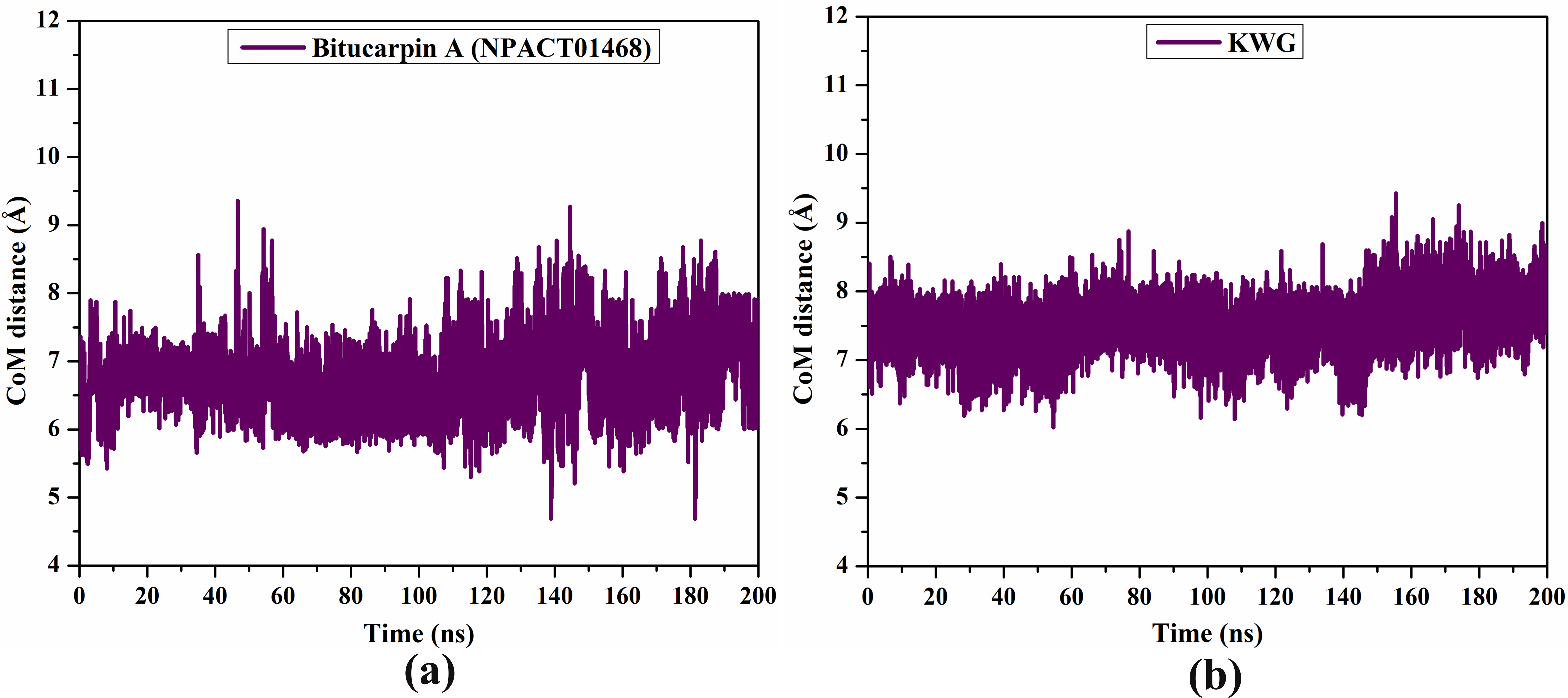


**Figure S3.** CoM distances of (a) bitucarpin A (NPACT01468) and (b) KWG and ASN519 of the EBNA1 over 200 ns MDS.

**Table S1.** The anticipated quick docking scores (in kcal/mol) for the NPACT compounds and KWG towards EBNA1.

| **No.** | **Compound Code** | **Docking Score (kcal/mol)** | **No.** | **Compound Code** | **Docking Score (kcal/mol)** | **No.** | **Compound Code** | **Docking Score (kcal/mol)** |
| --- | --- | --- | --- | --- | --- | --- | --- | --- |
|  | **KWG** | **–7.8** | 48 | NPACT01468 | –8.0 | 96 | NPACT01204 | –7.5 |
| 1 | NPACT00148 | –9.7 | 49 | NPACT00195 | –8.0 | 97 | NPACT00303 | –7.5 |
| 2 | NPACT01033 | –9.3 | 50 | NPACT01427 | –8.0 | 98 | NPACT00466 | –7.5 |
| 3 | NPACT00124 | –9.3 | 51 | NPACT00034 | –7.9 | 99 | NPACT00919 | –7.5 |
| 4 | NPACT01326 | –9.2 | 52 | NPACT01125 | –7.9 | 100 | NPACT01297 | –7.5 |
| 5 | NPACT01325 | –9.0 | 53 | NPACT00549 | –7.9 | 101 | NPACT01321 | –7.5 |
| 6 | NPACT00309 | –9.0 | 54 | NPACT01341 | –7.9 | 102 | NPACT01425 | –7.5 |
| 7 | NPACT01327 | –9.0 | 55 | NPACT00558 | –7.9 | 103 | NPACT00084 | –7.5 |
| 8 | NPACT00774 | –8.9 | 56 | NPACT00576 | –7.9 | 104 | NPACT01295 | –7.5 |
| 9 | NPACT01316 | –8.7 | 57 | NPACT00472 | –7.9 | 105 | NPACT00284 | –7.5 |
| 10 | NPACT00685 | –8.7 | 58 | NPACT01483 | –7.9 | 106 | NPACT01333 | –7.5 |
| 11 | NPACT00382 | –8.7 | 59 | NPACT01209 | –7.9 | 107 | NPACT01452 | –7.5 |
| 12 | NPACT00307 | –8.6 | 60 | NPACT00289 | –7.9 | 108 | NPACT00239 | –7.5 |
| 13 | NPACT01268 | –8.6 | 61 | NPACT01248 | –7.9 | 109 | NPACT00525 | –7.4 |
| 14 | NPACT00306 | –8.6 | 62 | NPACT00075 | –7.9 | 110 | NPACT01471 | –7.4 |
| 15 | NPACT01034 | –8.6 | 63 | NPACT00176 | –7.9 | 111 | NPACT01239 | –7.4 |
| 16 | NPACT01235 | –8.5 | 64 | NPACT01260 | –7.9 | 112 | NPACT01237 | –7.4 |
| 17 | NPACT01496 | –8.5 | 65 | NPACT01154 | –7.8 | 113 | NPACT01378 | –7.4 |
| 18 | NPACT00447 | –8.5 | 66 | NPACT00088 | –7.8 | 114 | NPACT00883 | –7.4 |
| 19 | NPACT00864 | –8.4 | 67 | NPACT00815 | –7.7 | 115 | NPACT01332 | –7.4 |
| 20 | NPACT00189 | –8.4 | 68 | NPACT00761 | –7.7 | 116 | NPACT00259 | –7.4 |
| 21 | NPACT01410 | –8.4 | 69 | NPACT01192 | –7.7 | 117 | NPACT00939 | –7.4 |
| 22 | NPACT01342 | –8.4 | 70 | NPACT01223 | –7.7 | 118 | NPACT00524 | –7.4 |
| 23 | NPACT01155 | –8.4 | 71 | NPACT00358 | –7.7 | 119 | NPACT01433 | –7.4 |
| 24 | NPACT01270 | –8.4 | 72 | NPACT00824 | –7.7 | 120 | NPACT00487 | –7.4 |
| 25 | NPACT00017 | –8.3 | 73 | NPACT01261 | –7.7 | 121 | NPACT00826 | –7.4 |
| 26 | NPACT00305 | –8.3 | 74 | NPACT01451 | –7.7 | 122 | NPACT00831 | –7.4 |
| 27 | NPACT00512 | –8.3 | 75 | NPACT00730 | –7.7 | 123 | NPACT00985 | –7.4 |
| 28 | NPACT01200 | –8.3 | 76 | NPACT00877 | –7.7 | 124 | NPACT00914 | –7.4 |
| 29 | NPACT00123 | –8.3 | 77 | NPACT00726 | –7.7 | 125 | NPACT00592 | –7.4 |
| 30 | NPACT00062 | –8.3 | 78 | NPACT00727 | –7.6 | 126 | NPACT01443 | –7.4 |
| 31 | NPACT01000 | –8.2 | 79 | NPACT00951 | –7.6 | 127 | NPACT01537 | –7.4 |
| 32 | NPACT01049 | –8.2 | 80 | NPACT00879 | –7.6 | 128 | NPACT01186 | –7.4 |
| 33 | NPACT00304 | –8.2 | 81 | NPACT01124 | –7.6 | 129 | NPACT01272 | –7.3 |
| 34 | NPACT01271 | –8.2 | 82 | NPACT00961 | –7.6 | 130 | NPACT01507 | –7.3 |
| 35 | NPACT01329 | –8.2 | 83 | NPACT00702 | –7.6 | 131 | NPACT00559 | –7.3 |
| 36 | NPACT01226 | –8.2 | 84 | NPACT01334 | –7.6 | 132 | NPACT00146 | –7.3 |
| 37 | NPACT00446 | –8.2 | 85 | NPACT01198 | –7.6 | 133 | NPACT00196 | –7.3 |
| 38 | NPACT01328 | –8.2 | 86 | NPACT00500 | –7.6 | 134 | NPACT00797 | –7.3 |
| 39 | NPACT01197 | –8.2 | 87 | NPACT01066 | –7.6 | 135 | NPACT00327 | –7.3 |
| 40 | NPACT00995 | –8.2 | 88 | NPACT00739 | –7.6 | 136 | NPACT00565 | –7.3 |
| 41 | NPACT00475 | –8.2 | 89 | NPACT01161 | –7.6 | 137 | NPACT01158 | –7.3 |
| 42 | NPACT00700 | –8.1 | 90 | NPACT00392 | –7.6 | 138 | NPACT00937 | –7.3 |
| 43 | NPACT00557 | –8.1 | 91 | NPACT00184 | –7.6 | 139 | NPACT01291 | –7.3 |
| 44 | NPACT00560 | –8.1 | 92 | NPACT00256 | –7.5 | 140 | NPACT00479 | –7.3 |
| 45 | NPACT00909 | –8.1 | 93 | NPACT01322 | –7.5 | 141 | NPACT00483 | –7.3 |
| 46 | NPACT01273 | –8.0 | 94 | NPACT00308 | –7.5 | 142 | NPACT00929 | –7.3 |
| 47 | NPACT00829 | –8.0 | 95 | NPACT01140 | –7.5 | 143 | NPACT01282 | –7.3 |

**Table S1.** *Continued*.

| **No.** | **Compound Code** | **Docking Score (kcal/mol)** | **No.** | **Compound Code** | **Docking Score (kcal/mol)** | **No.** | **Compound Code** | **Docking Score (kcal/mol)** |
| --- | --- | --- | --- | --- | --- | --- | --- | --- |
| 144 | NPACT00040 | –7.3 | 194 | NPACT00173 | –7.2 | 244 | NPACT01430 | –7.0 |
| 145 | NPACT00067 | –7.3 | 195 | NPACT00734 | –7.1 | 245 | NPACT01566 | –7.0 |
| 146 | NPACT00302 | –7.3 | 196 | NPACT01405 | –7.1 | 246 | NPACT00551 | –7.0 |
| 147 | NPACT00285 | –7.3 | 197 | NPACT01475 | –7.1 | 247 | NPACT01417 | –7.0 |
| 148 | NPACT00332 | –7.3 | 198 | NPACT00175 | –7.1 | 248 | NPACT00153 | –7.0 |
| 149 | NPACT00389 | –7.3 | 199 | NPACT00197 | –7.1 | 249 | NPACT00325 | –7.0 |
| 150 | NPACT01009 | –7.3 | 200 | NPACT00411 | –7.1 | 250 | NPACT00347 | –7.0 |
| 151 | NPACT00006 | –7.2 | 201 | NPACT01054 | –7.1 | 251 | NPACT00704 | –7.0 |
| 152 | NPACT00103 | –7.2 | 202 | NPACT01251 | –7.1 | 252 | NPACT01182 | –7.0 |
| 153 | NPACT00589 | –7.2 | 203 | NPACT00061 | –7.1 | 253 | NPACT01474 | –7.0 |
| 154 | NPACT00637 | –7.2 | 204 | NPACT00273 | –7.1 | 254 | NPACT01091 | –7.0 |
| 155 | NPACT01191 | –7.2 | 205 | NPACT00351 | –7.1 | 255 | NPACT01128 | –7.0 |
| 156 | NPACT01363 | –7.2 | 206 | NPACT01022 | –7.1 | 256 | NPACT01162 | –7.0 |
| 157 | NPACT00128 | –7.2 | 207 | NPACT01286 | –7.1 | 257 | NPACT00222 | –7.0 |
| 158 | NPACT00471 | –7.2 | 208 | NPACT00271 | –7.1 | 258 | NPACT00365 | –7.0 |
| 159 | NPACT00741 | –7.2 | 209 | NPACT00501 | –7.1 | 259 | NPACT01013 | –7.0 |
| 160 | NPACT01024 | –7.2 | 210 | NPACT00953 | –7.1 | 260 | NPACT01335 | –7.0 |
| 161 | NPACT00464 | –7.2 | 211 | NPACT01206 | –7.1 | 261 | NPACT01434 | –7.0 |
| 162 | NPACT00988 | –7.2 | 212 | NPACT01504 | –7.1 | 262 | NPACT00910 | –7.0 |
| 163 | NPACT01508 | –7.2 | 213 | NPACT00484 | –7.1 | 263 | NPACT01184 | –7.0 |
| 164 | NPACT00612 | –7.2 | 214 | NPACT01108 | –7.1 | 264 | NPACT00481 | –7.0 |
| 165 | NPACT01018 | –7.2 | 215 | NPACT01199 | –7.1 | 265 | NPACT00938 | –7.0 |
| 166 | NPACT01036 | –7.2 | 216 | NPACT01320 | –7.1 | 266 | NPACT01196 | –7.0 |
| 167 | NPACT00125 | –7.2 | 217 | NPACT01402 | –7.1 | 267 | NPACT01310 | –7.0 |
| 168 | NPACT00878 | –7.2 | 218 | NPACT01421 | –7.1 | 268 | NPACT01356 | –7.0 |
| 169 | NPACT00880 | –7.2 | 219 | NPACT01574 | –7.1 | 269 | NPACT00037 | –7.0 |
| 170 | NPACT00940 | –7.2 | 220 | NPACT00107 | –7.1 | 270 | NPACT00636 | –7.0 |
| 171 | NPACT01188 | –7.2 | 221 | NPACT00758 | –7.1 | 271 | NPACT00701 | –7.0 |
| 172 | NPACT00038 | –7.2 | 222 | NPACT01243 | –7.1 | 272 | NPACT01145 | –7.0 |
| 173 | NPACT00114 | –7.2 | 223 | NPACT00333 | –7.1 | 273 | NPACT00300 | –7.0 |
| 174 | NPACT01240 | –7.2 | 224 | NPACT00563 | –7.1 | 274 | NPACT00775 | –7.0 |
| 175 | NPACT00051 | –7.2 | 225 | NPACT00884 | –7.1 | 275 | NPACT00917 | –7.0 |
| 176 | NPACT00198 | –7.2 | 226 | NPACT01208 | –7.1 | 276 | NPACT00069 | –6.9 |
| 177 | NPACT00510 | –7.2 | 227 | NPACT00331 | –7.1 | 277 | NPACT00230 | –6.9 |
| 178 | NPACT01398 | –7.2 | 228 | NPACT00468 | –7.1 | 278 | NPACT00912 | –6.9 |
| 179 | NPACT00301 | –7.2 | 229 | NPACT00480 | –7.2 | 279 | NPACT01267 | –6.9 |
| 180 | NPACT01403 | –7.3 | 230 | NPACT00713 | –7.2 | 280 | NPACT01430 | –6.9 |
| 181 | NPACT01102 | –7.3 | 231 | NPACT01011 | –7.1 | 281 | NPACT00623 | –6.9 |
| 182 | NPACT00173 | –7.3 | 232 | NPACT01567 | –7.1 | 282 | NPACT01080 | –6.9 |
| 183 | NPACT00734 | –7.3 | 233 | NPACT00183 | –7.1 | 283 | NPACT01103 | –6.9 |
| 184 | NPACT00038 | –7.3 | 234 | NPACT00828 | –7.1 | 284 | NPACT01420 | –6.9 |
| 185 | NPACT00114 | –7.3 | 235 | NPACT01379 | –7.1 | 285 | NPACT01426 | –6.9 |
| 186 | NPACT01240 | –7.3 | 236 | NPACT00778 | –7.1 | 286 | NPACT01435 | –6.9 |
| 187 | NPACT00051 | –7.2 | 237 | NPACT00916 | –7.1 | 287 | NPACT00817 | –6.9 |
| 188 | NPACT00198 | –7.2 | 238 | NPACT00043 | –7.1 | 288 | NPACT01001 | –7.0 |
| 189 | NPACT00510 | –7.2 | 239 | NPACT00514 | –7.1 | 289 | NPACT01340 | –7.0 |
| 190 | NPACT01398 | –7.2 | 240 | NPACT00663 | –7.1 | 290 | NPACT00911 | –7.0 |
| 191 | NPACT00301 | –7.2 | 241 | NPACT00837 | –7.1 | 291 | NPACT01048 | –7.0 |
| 192 | NPACT01403 | –7.2 | 242 | NPACT00912 | –7.0 | 292 | NPACT01392 | –7.0 |
| 193 | NPACT01102 | –7.2 | 243 | NPACT01267 | –7.0 | 293 | NPACT01469 | –7.0 |

**Table S1.** *Continued*.

| **No.** | **Compound Code** | **Docking Score (kcal/mol)** | **No.** | **Compound Code** | **Docking Score (kcal/mol)** | **No.** | **Compound Code** | **Docking Score (kcal/mol)** |
| --- | --- | --- | --- | --- | --- | --- | --- | --- |
| 294 | NPACT01057 | –6.9 | 344 | NPACT01419 | –6.8 | 394 | NPACT00113 | –6.6 |
| 295 | NPACT01336 | –6.9 | 345 | NPACT01429 | –6.8 | 395 | NPACT01323 | –6.6 |
| 296 | NPACT01518 | –6.9 | 346 | NPACT00973 | –6.7 | 396 | NPACT01391 | –6.6 |
| 297 | NPACT00281 | –6.9 | 347 | NPACT01431 | –6.7 | 397 | NPACT01570 | –6.6 |
| 298 | NPACT00569 | –6.9 | 348 | NPACT01464 | –6.7 | 398 | NPACT00213 | –6.6 |
| 299 | NPACT01416 | –6.9 | 349 | NPACT00225 | –6.7 | 399 | NPACT00367 | –6.6 |
| 300 | NPACT00170 | –6.9 | 350 | NPACT01543 | –6.7 | 400 | NPACT00477 | –6.6 |
| 301 | NPACT00218 | –6.9 | 351 | NPACT00290 | –6.7 | 401 | NPACT00808 | –6.6 |
| 302 | NPACT00617 | –6.9 | 352 | NPACT00688 | –6.7 | 402 | NPACT00902 | –6.6 |
| 303 | NPACT00967 | –6.9 | 353 | NPACT00753 | –6.7 | 403 | NPACT01021 | –6.6 |
| 304 | NPACT01404 | –6.9 | 354 | NPACT01109 | –6.7 | 404 | NPACT01104 | –6.6 |
| 305 | NPACT01477 | –6.9 | 355 | NPACT00203 | –6.7 | 405 | NPACT01362 | –6.6 |
| 306 | NPACT00948 | –6.9 | 356 | NPACT00554 | –6.7 | 406 | NPACT00660 | –6.6 |
| 307 | NPACT01423 | –6.9 | 357 | NPACT01244 | –6.7 | 407 | NPACT01393 | –6.6 |
| 308 | NPACT00556 | –6.9 | 358 | NPACT00906 | –6.7 | 408 | NPACT00070 | –6.6 |
| 309 | NPACT00868 | –6.9 | 359 | NPACT01041 | –6.7 | 409 | NPACT00863 | –6.6 |
| 310 | NPACT01338 | –6.9 | 360 | NPACT01097 | –6.7 | 410 | NPACT01225 | –6.6 |
| 311 | NPACT00283 | –6.8 | 361 | NPACT01499 | –6.7 | 411 | NPACT00091 | –6.6 |
| 312 | NPACT00553 | –6.8 | 362 | NPACT00110 | –6.7 | 412 | NPACT00748 | –6.6 |
| 313 | NPACT00599 | –6.8 | 363 | NPACT00498 | –6.7 | 413 | NPACT01283 | –6.6 |
| 314 | NPACT00710 | –6.8 | 364 | NPACT00566 | –6.7 | 414 | NPACT01395 | –6.6 |
| 315 | NPACT00261 | –6.8 | 365 | NPACT00841 | –6.7 | 415 | NPACT01305 | –6.6 |
| 316 | NPACT00980 | –6.8 | 366 | NPACT00903 | –6.7 | 416 | NPACT01526 | –6.6 |
| 317 | NPACT00286 | –6.8 | 367 | NPACT01035 | –6.7 | 417 | NPACT00596 | –6.6 |
| 318 | NPACT00571 | –6.8 | 368 | NPACT01365 | –6.7 | 418 | NPACT00869 | –6.6 |
| 319 | NPACT00647 | –6.8 | 369 | NPACT01337 | –6.7 | 419 | NPACT01122 | –6.6 |
| 320 | NPACT00747 | –6.8 | 370 | NPACT00279 | –6.7 | 420 | NPACT01287 | –6.6 |
| 321 | NPACT00807 | –6.8 | 371 | NPACT00954 | –6.7 | 421 | NPACT01382 | –6.6 |
| 322 | NPACT00974 | –6.8 | 372 | NPACT01490 | –6.7 | 422 | NPACT01528 | –6.6 |
| 323 | NPACT00232 | –6.8 | 373 | NPACT00419 | –6.7 | 423 | NPACT00689 | –6.6 |
| 324 | NPACT00502 | –6.8 | 374 | NPACT00582 | –6.7 | 424 | NPACT00898 | –6.6 |
| 325 | NPACT00810 | –6.8 | 375 | NPACT00638 | –6.7 | 425 | NPACT00927 | –6.6 |
| 326 | NPACT01520 | –6.8 | 376 | NPACT00716 | –6.7 | 426 | NPACT00463 | –6.6 |
| 327 | NPACT00364 | –6.8 | 377 | NPACT01227 | –6.7 | 427 | NPACT01318 | –6.6 |
| 328 | NPACT00670 | –6.8 | 378 | NPACT00538 | –6.7 | 428 | NPACT01374 | –6.6 |
| 329 | NPACT01100 | –6.8 | 379 | NPACT00662 | –6.7 | 429 | NPACT00215 | –6.5 |
| 330 | NPACT01274 | –6.8 | 380 | NPACT01086 | –6.7 | 430 | NPACT00282 | –6.5 |
| 331 | NPACT00443 | –6.8 | 381 | NPACT01148 | –6.7 | 431 | NPACT00287 | –6.5 |
| 332 | NPACT00490 | –6.8 | 382 | NPACT01361 | –6.7 | 432 | NPACT00321 | –6.5 |
| 333 | NPACT00497 | –6.8 | 383 | NPACT01377 | –6.7 | 433 | NPACT00709 | –6.5 |
| 334 | NPACT01480 | –6.8 | 384 | NPACT01401 | –6.7 | 434 | NPACT00736 | –6.5 |
| 335 | NPACT01515 | –6.8 | 385 | NPACT01529 | –6.7 | 435 | NPACT00756 | –6.5 |
| 336 | NPACT00167 | –6.8 | 386 | NPACT00093 | –6.6 | 436 | NPACT00784 | –6.5 |
| 337 | NPACT00799 | –6.8 | 387 | NPACT00210 | –6.6 | 437 | NPACT01187 | –6.5 |
| 338 | NPACT01121 | –6.8 | 388 | NPACT00278 | –6.6 | 438 | NPACT01384 | –6.5 |
| 339 | NPACT01463 | –6.8 | 389 | NPACT00628 | –6.6 | 439 | NPACT01487 | –6.5 |
| 340 | NPACT01255 | –6.8 | 390 | NPACT00901 | –6.6 | 440 | NPACT01216 | –6.5 |
| 341 | NPACT01294 | –6.8 | 391 | NPACT01298 | –6.6 | 441 | NPACT01412 | –6.5 |
| 342 | NPACT01568 | –6.8 | 392 | NPACT01300 | –6.6 | 442 | NPACT01523 | –6.5 |
| 343 | NPACT00682 | –6.8 | 393 | NPACT01439 | –6.6 | 443 | NPACT01569 | –6.5 |

**Table S1.** *Continued*.

| **No.** | **Compound Code** | **Docking Score (kcal/mol)** | **No.** | **Compound Code** | **Docking Score (kcal/mol)** | **No.** | **Compound Code** | **Docking Score (kcal/mol)** |
| --- | --- | --- | --- | --- | --- | --- | --- | --- |
| 444 | NPACT00562 | –6.5 | 494 | NPACT00089 | –6.4 | 544 | NPACT00729 | –6.3 |
| 445 | NPACT00675 | –6.5 | 495 | NPACT00482 | –6.4 | 545 | NPACT00353 | –6.3 |
| 446 | NPACT00908 | –6.5 | 496 | NPACT01445 | –6.4 | 546 | NPACT00552 | –6.3 |
| 447 | NPACT01071 | –6.5 | 497 | NPACT01455 | –6.4 | 547 | NPACT01386 | –6.3 |
| 448 | NPACT01257 | –6.5 | 498 | NPACT01498 | –6.4 | 548 | NPACT01454 | –6.3 |
| 449 | NPACT01397 | –6.5 | 499 | NPACT00211 | –6.4 | 549 | NPACT00027 | –6.3 |
| 450 | NPACT00564 | –6.5 | 500 | NPACT00231 | –6.4 | 550 | NPACT00112 | –6.3 |
| 451 | NPACT00674 | –6.5 | 501 | NPACT01068 | –6.4 | 551 | NPACT00166 | –6.3 |
| 452 | NPACT00680 | –6.5 | 502 | NPACT01269 | –6.4 | 552 | NPACT00270 | –6.3 |
| 453 | NPACT00989 | –6.5 | 503 | NPACT01339 | –6.4 | 553 | NPACT00420 | –6.3 |
| 454 | NPACT01160 | –6.5 | 504 | NPACT00434 | –6.4 | 554 | NPACT00509 | –6.3 |
| 455 | NPACT01296 | –6.5 | 505 | NPACT01170 | –6.4 | 555 | NPACT00755 | –6.3 |
| 456 | NPACT01390 | –6.5 | 506 | NPACT00354 | –6.4 | 556 | NPACT00779 | –6.3 |
| 457 | NPACT01375 | –6.5 | 507 | NPACT00809 | –6.4 | 557 | NPACT01245 | –6.3 |
| 458 | NPACT01389 | –6.5 | 508 | NPACT01020 | –6.4 | 558 | NPACT01126 | –6.3 |
| 459 | NPACT01512 | –6.5 | 509 | NPACT01115 | –6.4 | 559 | NPACT01314 | –6.3 |
| 460 | NPACT00659 | –6.5 | 510 | NPACT00661 | –6.4 | 560 | NPACT01411 | –6.3 |
| 461 | NPACT00754 | –6.5 | 511 | NPACT00830 | –6.4 | 561 | NPACT01517 | –6.3 |
| 462 | NPACT00838 | –6.5 | 512 | NPACT01222 | –6.4 | 562 | NPACT00334 | –6.3 |
| 463 | NPACT01023 | –6.5 | 513 | NPACT00766 | –6.4 | 563 | NPACT00338 | –6.3 |
| 464 | NPACT01067 | –6.5 | 514 | NPACT00796 | –6.4 | 564 | NPACT00462 | –6.3 |
| 465 | NPACT01092 | –6.5 | 515 | NPACT01040 | –6.4 | 565 | NPACT00806 | –6.3 |
| 466 | NPACT00053 | –6.5 | 516 | NPACT01231 | –6.4 | 566 | NPACT00519 | –6.2 |
| 467 | NPACT00165 | –6.5 | 517 | NPACT01258 | –6.4 | 567 | NPACT00445 | –6.2 |
| 468 | NPACT00456 | –6.5 | 518 | NPACT00021 | –6.4 | 568 | NPACT00602 | –6.2 |
| 469 | NPACT00854 | –6.5 | 519 | NPACT00749 | –6.4 | 569 | NPACT01246 | –6.2 |
| 470 | NPACT00928 | –6.5 | 520 | NPACT00907 | –6.4 | 570 | NPACT00162 | –6.2 |
| 471 | NPACT01259 | –6.5 | 521 | NPACT00342 | –6.4 | 571 | NPACT00117 | –6.2 |
| 472 | NPACT01406 | –6.5 | 522 | NPACT00588 | –6.4 | 572 | NPACT00409 | –6.2 |
| 473 | NPACT01509 | –6.5 | 523 | NPACT00759 | –6.4 | 573 | NPACT01112 | –6.2 |
| 474 | NPACT00190 | –6.5 | 524 | NPACT00825 | –6.4 | 574 | NPACT01143 | –6.2 |
| 475 | NPACT00735 | –6.5 | 525 | NPACT01077 | –6.4 | 575 | NPACT01224 | –6.2 |
| 476 | NPACT01195 | –6.5 | 526 | NPACT00527 | –6.3 | 576 | NPACT01358 | –6.2 |
| 477 | NPACT01262 | –6.5 | 527 | NPACT00964 | –6.3 | 577 | NPACT00359 | –6.2 |
| 478 | NPACT01359 | –6.5 | 528 | NPACT01030 | –6.3 | 578 | NPACT01090 | –6.2 |
| 479 | NPACT00811 | –6.5 | 529 | NPACT01064 | –6.3 | 579 | NPACT01376 | –6.2 |
| 480 | NPACT00905 | –6.5 | 530 | NPACT00277 | –6.3 | 580 | NPACT01383 | –6.2 |
| 481 | NPACT01043 | –6.5 | 531 | NPACT00712 | –6.3 | 581 | NPACT01396 | –6.2 |
| 482 | NPACT00360 | –6.5 | 532 | NPACT01288 | –6.3 | 582 | NPACT01516 | –6.2 |
| 483 | NPACT00703 | –6.5 | 533 | NPACT00740 | –6.3 | 583 | NPACT00521 | –6.2 |
| 484 | NPACT00798 | –6.5 | 534 | NPACT00857 | –6.3 | 584 | NPACT00752 | –6.2 |
| 485 | NPACT01185 | –6.5 | 535 | NPACT00873 | –6.3 | 585 | NPACT00820 | –6.2 |
| 486 | NPACT01381 | –6.5 | 536 | NPACT01159 | –6.3 | 586 | NPACT00893 | –6.2 |
| 487 | NPACT01414 | –6.5 | 537 | NPACT01357 | –6.3 | 587 | NPACT01062 | –6.2 |
| 488 | NPACT00547 | –6.4 | 538 | NPACT00066 | –6.3 | 588 | NPACT01424 | –6.2 |
| 489 | NPACT01399 | –6.4 | 539 | NPACT00329 | –6.3 | 589 | NPACT01493 | –6.2 |
| 490 | NPACT00852 | –6.4 | 540 | NPACT01500 | –6.3 | 590 | NPACT01531 | –6.2 |
| 491 | NPACT01163 | –6.4 | 541 | NPACT00064 | –6.3 | 591 | NPACT00237 | –6.2 |
| 492 | NPACT01290 | –6.4 | 542 | NPACT00121 | –6.3 | 592 | NPACT00495 | –6.2 |
| 493 | NPACT01488 | –6.4 | 543 | NPACT00520 | –6.3 | 593 | NPACT00987 | –6.2 |

**Table S1.** *Continued*.

| **No.** | **Compound Code** | **Docking Score (kcal/mol)** | **No.** | **Compound Code** | **Docking Score (kcal/mol)** | **No.** | **Compound Code** | **Docking Score (kcal/mol)** |
| --- | --- | --- | --- | --- | --- | --- | --- | --- |
| 594 | NPACT01060 | –6.2 | 644 | NPACT00706 | –6.1 | 694 | NPACT01436 | –6.0 |
| 595 | NPACT01233 | –6.2 | 645 | NPACT01394 | –6.1 | 695 | NPACT00380 | –6.0 |
| 596 | NPACT00155 | –6.2 | 646 | NPACT00548 | –6.1 | 696 | NPACT00658 | –6.0 |
| 597 | NPACT00243 | –6.2 | 647 | NPACT00949 | –6.1 | 697 | NPACT00783 | –6.0 |
| 598 | NPACT00470 | –6.2 | 648 | NPACT01051 | –6.1 | 698 | NPACT01534 | –6.0 |
| 599 | NPACT00818 | –6.2 | 649 | NPACT01089 | –6.1 | 699 | NPACT00092 | –6.0 |
| 600 | NPACT00885 | –6.2 | 650 | NPACT01254 | –6.1 | 700 | NPACT00104 | –6.0 |
| 601 | NPACT00891 | –6.2 | 651 | NPACT00585 | –6.1 | 701 | NPACT00335 | –6.0 |
| 602 | NPACT01236 | –6.2 | 652 | NPACT00587 | –6.1 | 702 | NPACT00693 | –6.0 |
| 603 | NPACT01385 | –6.2 | 653 | NPACT00622 | –6.1 | 703 | NPACT00725 | –6.0 |
| 604 | NPACT00291 | –6.2 | 654 | NPACT00126 | –6.1 | 704 | NPACT01047 | –6.0 |
| 605 | NPACT00665 | –6.2 | 655 | NPACT01230 | –6.1 | 705 | NPACT01311 | –6.0 |
| 606 | NPACT00776 | –6.2 | 656 | NPACT01514 | –6.1 | 706 | NPACT01352 | –6.0 |
| 607 | NPACT00892 | –6.2 | 657 | NPACT00002 | –6.1 | 707 | NPACT01453 | –6.0 |
| 608 | NPACT01087 | –6.2 | 658 | NPACT00193 | –6.1 | 708 | NPACT00090 | –6.0 |
| 609 | NPACT01133 | –6.2 | 659 | NPACT00324 | –6.1 | 709 | NPACT00227 | –6.0 |
| 610 | NPACT00595 | –6.2 | 660 | NPACT01144 | –6.1 | 710 | NPACT00373 | –6.0 |
| 611 | NPACT00613 | –6.2 | 661 | NPACT00008 | –6.0 | 711 | NPACT00352 | –5.9 |
| 612 | NPACT00633 | –6.2 | 662 | NPACT00220 | –6.0 | 712 | NPACT00577 | –5.9 |
| 613 | NPACT00698 | –6.2 | 663 | NPACT00311 | –6.0 | 713 | NPACT00855 | –5.9 |
| 614 | NPACT00969 | –6.2 | 664 | NPACT00460 | –6.0 | 714 | NPACT00856 | –5.9 |
| 615 | NPACT01215 | –6.2 | 665 | NPACT00733 | –6.0 | 715 | NPACT00887 | –5.9 |
| 616 | NPACT01461 | –6.2 | 666 | NPACT00764 | –6.0 | 716 | NPACT00922 | –5.9 |
| 617 | NPACT00867 | –6.1 | 667 | NPACT00346 | –6.0 | 717 | NPACT00944 | –5.9 |
| 618 | NPACT01069 | –6.1 | 668 | NPACT01299 | –6.0 | 718 | NPACT01074 | –5.9 |
| 619 | NPACT01098 | –6.1 | 669 | NPACT01541 | –6.0 | 719 | NPACT01247 | –5.9 |
| 620 | NPACT01400 | –6.1 | 670 | NPACT00171 | –6.0 | 720 | NPACT01364 | –5.9 |
| 621 | NPACT00476 | –6.1 | 671 | NPACT00399 | –6.0 | 721 | NPACT00149 | –5.9 |
| 622 | NPACT01422 | –6.1 | 672 | NPACT00426 | –6.0 | 722 | NPACT00242 | –5.9 |
| 623 | NPACT01466 | –6.1 | 673 | NPACT01256 | –6.0 | 723 | NPACT00413 | –5.9 |
| 624 | NPACT01571 | –6.1 | 674 | NPACT01554 | –6.0 | 724 | NPACT00750 | –5.9 |
| 625 | NPACT00201 | –6.1 | 675 | NPACT00328 | –6.0 | 725 | NPACT00859 | –5.9 |
| 626 | NPACT00202 | –6.1 | 676 | NPACT00605 | –6.0 | 726 | NPACT01441 | –5.9 |
| 627 | NPACT00219 | –6.1 | 677 | NPACT00611 | –6.0 | 727 | NPACT00012 | –5.9 |
| 628 | NPACT00412 | –6.1 | 678 | NPACT00678 | –6.0 | 728 | NPACT01348 | –5.9 |
| 629 | NPACT00770 | –6.1 | 679 | NPACT01450 | –6.0 | 729 | NPACT00226 | –5.9 |
| 630 | NPACT00771 | –6.1 | 680 | NPACT00236 | –6.0 | 730 | NPACT00245 | –5.9 |
| 631 | NPACT00842 | –6.1 | 681 | NPACT00804 | –6.0 | 731 | NPACT00816 | –5.9 |
| 632 | NPACT01070 | –6.1 | 682 | NPACT00822 | –6.0 | 732 | NPACT00860 | –5.9 |
| 633 | NPACT00570 | –6.1 | 683 | NPACT01217 | –6.0 | 733 | NPACT00943 | –5.9 |
| 634 | NPACT01317 | –6.1 | 684 | NPACT00111 | –6.0 | 734 | NPACT01211 | –5.9 |
| 635 | NPACT00046 | –6.1 | 685 | NPACT00129 | –6.0 | 735 | NPACT01129 | –5.9 |
| 636 | NPACT00178 | –6.1 | 686 | NPACT00590 | –6.0 | 736 | NPACT01202 | –5.9 |
| 637 | NPACT00378 | –6.1 | 687 | NPACT01099 | –6.0 | 737 | NPACT01347 | –5.9 |
| 638 | NPACT01447 | –6.1 | 688 | NPACT01407 | –6.0 | 738 | NPACT01513 | –5.9 |
| 639 | NPACT00096 | –6.1 | 689 | NPACT00517 | –6.0 | 739 | NPACT00491 | –5.9 |
| 640 | NPACT00228 | –6.1 | 690 | NPACT00526 | –6.0 | 740 | NPACT00882 | –5.9 |
| 641 | NPACT00402 | –6.1 | 691 | NPACT00697 | –6.0 | 741 | NPACT01180 | –5.9 |
| 642 | NPACT00528 | –6.1 | 692 | NPACT00827 | –6.0 | 742 | NPACT01214 | –5.9 |
| 643 | NPACT00671 | –6.1 | 693 | NPACT01063 | –6.0 | 743 | NPACT00065 | –5.9 |

**Table S1.** *Continued*.

| **No.** | **Compound Code** | **Docking Score (kcal/mol)** | **No.** | **Compound Code** | **Docking Score (kcal/mol)** | **No.** | **Compound Code** | **Docking Score (kcal/mol)** |
| --- | --- | --- | --- | --- | --- | --- | --- | --- |
| 744 | NPACT00181 | –5.9 | 794 | NPACT01207 | –5.7 | 844 | NPACT00872 | –5.6 |
| 745 | NPACT01353 | –5.9 | 795 | NPACT01472 | –5.7 | 845 | NPACT01324 | –5.6 |
| 746 | NPACT01456 | –5.9 | 796 | NPACT01479 | –5.7 | 846 | NPACT01497 | –5.6 |
| 747 | NPACT00151 | –5.9 | 797 | NPACT01076 | –5.7 | 847 | NPACT00934 | –5.6 |
| 748 | NPACT00398 | –5.9 | 798 | NPACT00150 | –5.7 | 848 | NPACT01082 | –5.6 |
| 749 | NPACT00621 | –5.9 | 799 | NPACT00572 | –5.7 | 849 | NPACT00039 | –5.5 |
| 750 | NPACT00970 | –5.9 | 800 | NPACT00586 | –5.7 | 850 | NPACT00609 | –5.5 |
| 751 | NPACT01428 | –5.9 | 801 | NPACT01117 | –5.7 | 851 | NPACT00876 | –5.5 |
| 752 | NPACT01432 | –5.9 | 802 | NPACT00233 | –5.7 | 852 | NPACT00888 | –5.5 |
| 753 | NPACT00169 | –5.9 | 803 | NPACT00813 | –5.7 | 853 | NPACT01019 | –5.5 |
| 754 | NPACT00821 | –5.9 | 804 | NPACT00322 | –5.7 | 854 | NPACT01437 | –5.5 |
| 755 | NPACT01052 | –5.9 | 805 | NPACT01078 | –5.7 | 855 | NPACT00028 | –5.5 |
| 756 | NPACT00191 | –5.9 | 806 | NPACT01302 | –5.7 | 856 | NPACT00802 | –5.5 |
| 757 | NPACT01106 | –5.9 | 807 | NPACT00185 | –5.7 | 857 | NPACT01059 | –5.5 |
| 758 | NPACT00045 | –5.8 | 808 | NPACT00640 | –5.7 | 858 | NPACT01010 | –5.5 |
| 759 | NPACT00686 | –5.8 | 809 | NPACT00643 | –5.7 | 859 | NPACT00410 | –5.5 |
| 760 | NPACT00019 | –5.8 | 810 | NPACT01413 | –5.7 | 860 | NPACT00666 | –5.5 |
| 761 | NPACT00025 | –5.8 | 811 | NPACT00581 | –5.6 | 861 | NPACT00942 | –5.5 |
| 762 | NPACT00751 | –5.8 | 812 | NPACT01075 | –5.6 | 862 | NPACT01039 | –5.5 |
| 763 | NPACT01084 | –5.8 | 813 | NPACT01094 | –5.6 | 863 | NPACT01438 | –5.5 |
| 764 | NPACT00372 | –5.8 | 814 | NPACT00077 | –5.6 | 864 | NPACT01081 | –5.5 |
| 765 | NPACT00794 | –5.8 | 815 | NPACT00254 | –5.6 | 865 | NPACT00603 | –5.5 |
| 766 | NPACT00861 | –5.8 | 816 | NPACT00257 | –5.6 | 866 | NPACT00036 | –5.5 |
| 767 | NPACT00965 | –5.8 | 817 | NPACT00363 | –5.6 | 867 | NPACT01085 | –5.5 |
| 768 | NPACT01050 | –5.8 | 818 | NPACT00390 | –5.6 | 868 | NPACT01482 | –5.5 |
| 769 | NPACT01176 | –5.8 | 819 | NPACT00579 | –5.6 | 869 | NPACT00098 | –5.5 |
| 770 | NPACT01446 | –5.8 | 820 | NPACT00763 | –5.6 | 870 | NPACT00428 | –5.5 |
| 771 | NPACT00068 | –5.8 | 821 | NPACT00915 | –5.6 | 871 | NPACT01494 | –5.5 |
| 772 | NPACT00494 | –5.8 | 822 | NPACT01056 | –5.6 | 872 | NPACT00478 | –5.5 |
| 773 | NPACT00511 | –5.8 | 823 | NPACT00094 | –5.6 | 873 | NPACT00030 | –5.5 |
| 774 | NPACT01228 | –5.8 | 824 | NPACT00217 | –5.6 | 874 | NPACT00161 | –5.5 |
| 775 | NPACT00095 | –5.8 | 825 | NPACT00336 | –5.6 | 875 | NPACT00667 | –5.5 |
| 776 | NPACT01304 | –5.8 | 826 | NPACT00344 | –5.6 | 876 | NPACT00870 | –5.5 |
| 777 | NPACT00594 | –5.8 | 827 | NPACT01229 | –5.6 | 877 | NPACT00250 | –5.4 |
| 778 | NPACT00026 | –5.8 | 828 | NPACT00085 | –5.6 | 878 | NPACT00962 | –5.4 |
| 779 | NPACT00337 | –5.8 | 829 | NPACT00312 | –5.6 | 879 | NPACT01183 | –5.4 |
| 780 | NPACT00760 | –5.8 | 830 | NPACT00451 | –5.6 | 880 | NPACT00152 | –5.4 |
| 781 | NPACT00986 | –5.8 | 831 | NPACT00642 | –5.6 | 881 | NPACT00941 | –5.4 |
| 782 | NPACT01459 | –5.8 | 832 | NPACT01201 | –5.6 | 882 | NPACT01330 | –5.4 |
| 783 | NPACT00179 | –5.8 | 833 | NPACT00992 | –5.6 | 883 | NPACT00280 | –5.4 |
| 784 | NPACT00676 | –5.8 | 834 | NPACT01281 | –5.6 | 884 | NPACT00994 | –5.4 |
| 785 | NPACT01053 | –5.8 | 835 | NPACT00122 | –5.6 | 885 | NPACT01037 | –5.4 |
| 786 | NPACT00272 | –5.8 | 836 | NPACT00913 | –5.6 | 886 | NPACT00414 | –5.4 |
| 787 | NPACT00800 | –5.8 | 837 | NPACT01164 | –5.6 | 887 | NPACT01535 | –5.4 |
| 788 | NPACT00996 | –5.8 | 838 | NPACT01481 | –5.6 | 888 | NPACT01134 | –5.4 |
| 789 | NPACT00182 | –5.8 | 839 | NPACT00452 | –5.6 | 889 | NPACT00690 | –5.4 |
| 790 | NPACT00644 | –5.8 | 840 | NPACT00777 | –5.6 | 890 | NPACT00918 | –5.4 |
| 791 | NPACT00886 | –5.8 | 841 | NPACT00684 | –5.6 | 891 | NPACT01540 | –5.4 |
| 792 | NPACT00561 | –5.7 | 842 | NPACT00803 | –5.6 | 892 | NPACT00188 | –5.4 |
| 793 | NPACT01415 | –5.7 | 843 | NPACT00839 | –5.6 | 893 | NPACT00391 | –5.4 |

**Table S1.** *Continued*.

| **No.** | **Compound Code** | **Docking Score (kcal/mol)** | **No.** | **Compound Code** | **Docking Score (kcal/mol)** | **No.** | **Compound Code** | **Docking Score (kcal/mol)** |
| --- | --- | --- | --- | --- | --- | --- | --- | --- |
| 894 | NPACT00732 | –5.4 | 944 | NPACT00168 | –5.2 | 994 | NPACT01510 | –5.0 |
| 895 | NPACT00805 | –5.4 | 945 | NPACT01003 | –5.2 | 995 | NPACT01360 | –5.0 |
| 896 | NPACT01055 | –5.4 | 946 | NPACT01044 | –5.2 | 996 | NPACT00743 | –5.0 |
| 897 | NPACT00601 | –5.4 | 947 | NPACT01101 | –5.2 | 997 | NPACT00896 | –5.0 |
| 898 | NPACT00608 | –5.3 | 948 | NPACT01219 | –5.2 | 998 | NPACT00246 | –5.0 |
| 899 | NPACT00790 | –5.3 | 949 | NPACT00450 | –5.2 | 999 | NPACT00977 | –5.0 |
| 900 | NPACT01127 | –5.3 | 950 | NPACT00981 | –5.2 | 1000 | NPACT00108 | –5.0 |
| 901 | NPACT00194 | –5.3 | 951 | NPACT00018 | –5.2 | 1001 | NPACT00376 | –5.0 |
| 902 | NPACT00350 | –5.3 | 952 | NPACT00695 | –5.2 | 1002 | NPACT00492 | –5.0 |
| 903 | NPACT00388 | –5.3 | 953 | NPACT01210 | –5.2 | 1003 | NPACT00851 | –5.0 |
| 904 | NPACT00405 | –5.3 | 954 | NPACT01491 | –5.2 | 1004 | NPACT00020 | –5.0 |
| 905 | NPACT00506 | –5.3 | 955 | NPACT00699 | –5.1 | 1005 | NPACT00341 | –5.0 |
| 906 | NPACT00626 | –5.3 | 956 | NPACT00920 | –5.1 | 1006 | NPACT00433 | –5.0 |
| 907 | NPACT00641 | –5.3 | 957 | NPACT00377 | –5.1 | 1007 | NPACT00458 | –5.0 |
| 908 | NPACT01093 | –5.3 | 958 | NPACT00453 | –5.1 | 1008 | NPACT00041 | –4.9 |
| 909 | NPACT01132 | –5.3 | 959 | NPACT01538 | –5.1 | 1009 | NPACT00899 | –4.9 |
| 910 | NPACT00042 | –5.3 | 960 | NPACT00801 | –5.1 | 1010 | NPACT01147 | –4.9 |
| 911 | NPACT00679 | –5.3 | 961 | NPACT00240 | –5.1 | 1011 | NPACT01460 | –4.9 |
| 912 | NPACT00583 | –5.3 | 962 | NPACT01476 | –5.1 | 1012 | NPACT00348 | –4.9 |
| 913 | NPACT00163 | –5.3 | 963 | NPACT01119 | –5.1 | 1013 | NPACT00610 | –4.9 |
| 914 | NPACT00323 | –5.3 | 964 | NPACT00486 | –5.1 | 1014 | NPACT01168 | –4.9 |
| 915 | NPACT00326 | –5.3 | 965 | NPACT00767 | –5.1 | 1015 | NPACT01408 | –4.9 |
| 916 | NPACT00773 | –5.3 | 966 | NPACT01017 | –5.1 | 1016 | NPACT00469 | –4.9 |
| 917 | NPACT00979 | –5.3 | 967 | NPACT01238 | –5.1 | 1017 | NPACT00711 | –4.9 |
| 918 | NPACT01354 | –5.3 | 968 | NPACT00255 | –5.1 | 1018 | NPACT01542 | –4.9 |
| 919 | NPACT00615 | –5.3 | 969 | NPACT00362 | –5.1 | 1019 | NPACT00001 | –4.9 |
| 920 | NPACT00639 | –5.3 | 970 | NPACT00415 | –5.1 | 1020 | NPACT00317 | –4.9 |
| 921 | NPACT00947 | –5.3 | 971 | NPACT00361 | –5.1 | 1021 | NPACT00313 | –4.9 |
| 922 | NPACT00005 | –5.3 | 972 | NPACT00574 | –5.1 | 1022 | NPACT00865 | –4.9 |
| 923 | NPACT00407 | –5.3 | 973 | NPACT00792 | –5.1 | 1023 | NPACT00708 | –4.9 |
| 924 | NPACT01467 | –5.3 | 974 | NPACT00997 | –5.1 | 1024 | NPACT01114 | –4.9 |
| 925 | NPACT00288 | –5.3 | 975 | NPACT01008 | –5.1 | 1025 | NPACT01502 | –4.9 |
| 926 | NPACT01343 | –5.3 | 976 | NPACT01289 | –5.1 | 1026 | NPACT00252 | –4.9 |
| 927 | NPACT00156 | –5.3 | 977 | NPACT00102 | –5.1 | 1027 | NPACT00515 | –4.9 |
| 928 | NPACT01553 | –5.3 | 978 | NPACT00120 | –5.1 | 1028 | NPACT00687 | –4.9 |
| 929 | NPACT01303 | –5.2 | 979 | NPACT00648 | –5.1 | 1029 | NPACT00871 | –4.9 |
| 930 | NPACT00651 | –5.2 | 980 | NPACT01448 | –5.1 | 1030 | NPACT00900 | –4.9 |
| 931 | NPACT01141 | –5.2 | 981 | NPACT00007 | –5.0 | 1031 | NPACT00998 | –4.9 |
| 932 | NPACT00668 | –5.2 | 982 | NPACT00976 | –5.0 | 1032 | NPACT01116 | –4.9 |
| 933 | NPACT00791 | –5.2 | 983 | NPACT01234 | –5.0 | 1033 | NPACT01175 | –4.9 |
| 934 | NPACT00955 | –5.2 | 984 | NPACT00580 | –5.0 | 1034 | NPACT00115 | –4.9 |
| 935 | NPACT00850 | –5.2 | 985 | NPACT00768 | –5.0 | 1035 | NPACT00518 | –4.9 |
| 936 | NPACT01146 | –5.2 | 986 | NPACT00819 | –5.0 | 1036 | NPACT01088 | –4.9 |
| 937 | NPACT00593 | –5.2 | 987 | NPACT00172 | –5.0 | 1037 | NPACT01253 | –4.9 |
| 938 | NPACT00930 | –5.2 | 988 | NPACT00397 | –5.0 | 1038 | NPACT01174 | –4.9 |
| 939 | NPACT00192 | –5.2 | 989 | NPACT00449 | –5.0 | 1039 | NPACT01265 | –4.8 |
| 940 | NPACT00606 | –5.2 | 990 | NPACT00795 | –5.0 | 1040 | NPACT01355 | –4.8 |
| 941 | NPACT00629 | –5.2 | 991 | NPACT00604 | –5.0 | 1041 | NPACT01440 | –4.8 |
| 942 | NPACT00683 | –5.2 | 992 | NPACT01004 | –5.0 | 1042 | NPACT00978 | –4.8 |
| 943 | NPACT01331 | –5.2 | 993 | NPACT01349 | –5.0 | 1043 | NPACT01264 | –4.8 |

**Table S1.** *Continued*.

| **No.** | **Compound Code** | **Docking Score (kcal/mol)** | **No.** | **Compound Code** | **Docking Score (kcal/mol)** | **No.** | **Compound Code** | **Docking Score (kcal/mol)** |
| --- | --- | --- | --- | --- | --- | --- | --- | --- |
| 1044 | NPACT01212 | –4.8 | 1094 | NPACT01470 | –4.5 | 1144 | NPACT00722 | –4.2 |
| 1045 | NPACT01275 | –4.8 | 1095 | NPACT00127 | –4.5 | 1145 | NPACT01150 | –4.2 |
| 1046 | NPACT00204 | –4.8 | 1096 | NPACT00715 | –4.5 | 1146 | NPACT00721 | –4.2 |
| 1047 | NPACT00705 | –4.8 | 1097 | NPACT00932 | –4.5 | 1147 | NPACT00921 | –4.2 |
| 1048 | NPACT00187 | –4.8 | 1098 | NPACT01113 | –4.5 | 1148 | NPACT00432 | –4.1 |
| 1049 | NPACT00238 | –4.8 | 1099 | NPACT00048 | –4.5 | 1149 | NPACT01250 | –4.1 |
| 1050 | NPACT00366 | –4.8 | 1100 | NPACT01193 | –4.5 | 1150 | NPACT00314 | –4.1 |
| 1051 | NPACT00421 | –4.8 | 1101 | NPACT01276 | –4.5 | 1151 | NPACT00248 | –4.1 |
| 1052 | NPACT00275 | –4.8 | 1102 | NPACT00485 | –4.5 | 1152 | NPACT00862 | –4.1 |
| 1053 | NPACT00656 | –4.8 | 1103 | NPACT00567 | –4.5 | 1153 | NPACT00442 | –4.1 |
| 1054 | NPACT01006 | –4.7 | 1104 | NPACT00315 | –4.4 | 1154 | NPACT00442 | –4.1 |
| 1055 | NPACT01486 | –4.7 | 1105 | NPACT00424 | –4.4 | 1155 | NPACT00542 | –4.1 |
| 1056 | NPACT00097 | –4.7 | 1106 | NPACT01072 | –4.4 | 1156 | NPACT01105 | –4.1 |
| 1057 | NPACT00431 | –4.7 | 1107 | NPACT00400 | –4.4 | 1157 | NPACT00786 | –4.1 |
| 1058 | NPACT00607 | –4.7 | 1108 | NPACT00536 | –4.4 | 1158 | NPACT01279 | –4.1 |
| 1059 | NPACT00975 | –4.7 | 1109 | NPACT01167 | –4.4 | 1159 | NPACT01409 | –4.1 |
| 1060 | NPACT00971 | –4.7 | 1110 | NPACT01079 | –4.4 | 1160 | NPACT00555 | –4.1 |
| 1061 | NPACT00550 | –4.7 | 1111 | NPACT00116 | –4.4 | 1161 | NPACT00832 | –4.1 |
| 1062 | NPACT00627 | –4.7 | 1112 | NPACT00212 | –4.4 | 1162 | NPACT01058 | –4.1 |
| 1063 | NPACT00793 | –4.7 | 1113 | NPACT00343 | –4.4 | 1163 | NPACT01532 | –4.1 |
| 1064 | NPACT00029 | –4.7 | 1114 | NPACT00316 | –4.4 | 1164 | NPACT01536 | –4.1 |
| 1065 | NPACT01511 | –4.7 | 1115 | NPACT00132 | –4.4 | 1165 | NPACT00154 | –4.0 |
| 1066 | NPACT00106 | –4.7 | 1116 | NPACT00866 | –4.4 | 1166 | NPACT00833 | –4.0 |
| 1067 | NPACT00131 | –4.7 | 1117 | NPACT01506 | –4.4 | 1167 | NPACT00982 | –4.0 |
| 1068 | NPACT00186 | –4.7 | 1118 | NPACT01544 | –4.4 | 1168 | NPACT01319 | –4.0 |
| 1069 | NPACT00320 | –4.7 | 1119 | NPACT00345 | –4.4 | 1169 | NPACT01137 | –4.0 |
| 1070 | NPACT00375 | –4.7 | 1120 | NPACT00056 | –4.3 | 1170 | NPACT00295 | –4.0 |
| 1071 | NPACT00454 | –4.7 | 1121 | NPACT00105 | –4.3 | 1171 | NPACT00384 | –4.0 |
| 1072 | NPACT00840 | –4.7 | 1122 | NPACT00664 | –4.3 | 1172 | NPACT00430 | –4.0 |
| 1073 | NPACT00513 | –4.6 | 1123 | NPACT00349 | –4.3 | 1173 | NPACT00442 | –4.0 |
| 1074 | NPACT00692 | –4.6 | 1124 | NPACT00654 | –4.3 | 1174 | NPACT01485 | –4.0 |
| 1075 | NPACT00423 | –4.6 | 1125 | NPACT00789 | –4.3 | 1175 | NPACT01278 | –4.0 |
| 1076 | NPACT00578 | –4.6 | 1126 | NPACT00370 | –4.3 | 1176 | NPACT01345 | –4.0 |
| 1077 | NPACT00669 | –4.6 | 1127 | NPACT01372 | –4.3 | 1177 | NPACT00714 | –4.0 |
| 1078 | NPACT00260 | –4.6 | 1128 | NPACT00504 | –4.3 | 1178 | NPACT00897 | –4.0 |
| 1079 | NPACT01046 | –4.6 | 1129 | NPACT00022 | –4.3 | 1179 | NPACT01139 | –4.0 |
| 1080 | NPACT00299 | –4.6 | 1130 | NPACT00339 | –4.3 | 1180 | NPACT00057 | –3.9 |
| 1081 | NPACT00568 | –4.6 | 1131 | NPACT01083 | –4.3 | 1181 | NPACT01280 | –3.9 |
| 1082 | NPACT00499 | –4.6 | 1132 | NPACT00677 | –4.2 | 1182 | NPACT00267 | –3.9 |
| 1083 | NPACT00401 | –4.6 | 1133 | NPACT01156 | –4.2 | 1183 | NPACT00383 | –3.9 |
| 1084 | NPACT00991 | –4.6 | 1134 | NPACT00055 | –4.2 | 1184 | NPACT00724 | –3.9 |
| 1085 | NPACT01179 | –4.6 | 1135 | NPACT00076 | –4.2 | 1185 | NPACT01284 | –3.9 |
| 1086 | NPACT00244 | –4.6 | 1136 | NPACT00158 | –4.2 | 1186 | NPACT00474 | –3.9 |
| 1087 | NPACT00823 | –4.6 | 1137 | NPACT00221 | –4.2 | 1187 | NPACT00645 | –3.9 |
| 1088 | NPACT01380 | –4.6 | 1138 | NPACT00923 | –4.2 | 1188 | NPACT00933 | –3.9 |
| 1089 | NPACT00049 | –4.6 | 1139 | NPACT00396 | –4.2 | 1189 | NPACT00276 | –3.9 |
| 1090 | NPACT00422 | –4.6 | 1140 | NPACT00772 | –4.2 | 1190 | NPACT00591 | –3.9 |
| 1091 | NPACT00385 | –4.5 | 1141 | NPACT00836 | –4.2 | 1191 | NPACT00356 | –3.9 |
| 1092 | NPACT00845 | –4.5 | 1142 | NPACT00881 | –4.2 | 1192 | NPACT00625 | –3.9 |
| 1093 | NPACT00080 | –4.5 | 1143 | NPACT01563 | –4.2 | 1193 | NPACT00457 | –3.9 |

**Table S1.** *Continued*.

| **No.** | **Compound Code** | **Docking Score (kcal/mol)** | **No.** | **Compound Code** | **Docking Score (kcal/mol)** | **No.** | **Compound Code** | **Docking Score (kcal/mol)** |
| --- | --- | --- | --- | --- | --- | --- | --- | --- |
| 1194 | NPACT00614 | –3.9 | 1244 | NPACT00003 | –3.3 | 1294 | NPACT00785 | –2.6 |
| 1195 | NPACT01521 | –3.9 | 1245 | NPACT00133 | –3.3 | 1295 | NPACT01194 | –2.6 |
| 1196 | NPACT00436 | –3.8 | 1246 | NPACT00894 | –3.3 | 1296 | NPACT00597 | –2.6 |
| 1197 | NPACT00635 | –3.8 | 1247 | NPACT01177 | –3.3 | 1297 | NPACT01107 | –2.6 |
| 1198 | NPACT00101 | –3.8 | 1248 | NPACT00032 | –3.3 | 1298 | NPACT00634 | –2.6 |
| 1199 | NPACT01344 | –3.8 | 1249 | NPACT00387 | –3.3 | 1299 | NPACT00957 | –2.6 |
| 1200 | NPACT00205 | –3.8 | 1250 | NPACT00757 | –3.2 | 1300 | NPACT00788 | –2.5 |
| 1201 | NPACT00429 | –3.8 | 1251 | NPACT00072 | –3.2 | 1301 | NPACT00159 | –2.5 |
| 1202 | NPACT01218 | –3.8 | 1252 | NPACT00931 | –3.2 | 1302 | NPACT00925 | –2.5 |
| 1203 | NPACT00118 | –3.8 | 1253 | NPACT00078 | –3.2 | 1303 | NPACT01171 | –2.5 |
| 1204 | NPACT00694 | –3.8 | 1254 | NPACT00403 | –3.2 | 1304 | NPACT00082 | –2.5 |
| 1205 | NPACT00147 | –3.8 | 1255 | NPACT00507 | –3.2 | 1305 | NPACT01473 | –2.4 |
| 1206 | NPACT00229 | –3.8 | 1256 | NPACT00461 | –3.2 | 1306 | NPACT01530 | –2.4 |
| 1207 | NPACT00224 | –3.7 | 1257 | NPACT01442 | –3.2 | 1307 | NPACT00059 | –2.3 |
| 1208 | NPACT00368 | –3.7 | 1258 | NPACT00834 | –3.1 | 1308 | NPACT00265 | –2.3 |
| 1209 | NPACT00086 | –3.7 | 1259 | NPACT01373 | –3.1 | 1309 | NPACT01136 | –2.3 |
| 1210 | NPACT00174 | –3.7 | 1260 | NPACT00620 | –3.1 | 1310 | NPACT01444 | –2.3 |
| 1211 | NPACT01130 | –3.7 | 1261 | NPACT01573 | –3.1 | 1311 | NPACT01138 | –2.2 |
| 1212 | NPACT01522 | –3.7 | 1262 | NPACT01293 | –3.1 | 1312 | NPACT01501 | –2.2 |
| 1213 | NPACT00959 | –3.7 | 1263 | NPACT00691 | –3.1 | 1313 | NPACT01458 | –2.1 |
| 1214 | NPACT01149 | –3.7 | 1264 | NPACT00993 | –3.1 | 1314 | NPACT00143 | –2.1 |
| 1215 | NPACT01152 | –3.7 | 1265 | NPACT01173 | –3.1 | 1315 | NPACT00060 | –2.1 |
| 1216 | NPACT01539 | –3.7 | 1266 | NPACT00177 | –3.1 | 1316 | NPACT00310 | –2.1 |
| 1217 | NPACT00890 | –3.7 | 1267 | NPACT01308 | –3.0 | 1317 | NPACT01120 | –2.1 |
| 1218 | NPACT00035 | –3.7 | 1268 | NPACT01203 | –3.0 | 1318 | NPACT00135 | –2.0 |
| 1219 | NPACT01178 | –3.7 | 1269 | NPACT01190 | –3.0 | 1319 | NPACT00058 | –2.0 |
| 1220 | NPACT00652 | –3.6 | 1270 | NPACT01142 | –3.0 | 1320 | NPACT00406 | –2.0 |
| 1221 | NPACT00054 | –3.6 | 1271 | NPACT00293 | –3.0 | 1321 | NPACT00787 | –2.0 |
| 1222 | NPACT00031 | –3.6 | 1272 | NPACT00488 | –3.0 | 1322 | NPACT00737 | –2.0 |
| 1223 | NPACT01449 | –3.6 | 1273 | NPACT01151 | –2.9 | 1323 | NPACT00234 | –1.9 |
| 1224 | NPACT00087 | –3.6 | 1274 | NPACT00448 | –2.9 | 1324 | NPACT00769 | –1.9 |
| 1225 | NPACT01118 | –3.6 | 1275 | NPACT00946 | –2.8 | 1325 | NPACT00835 | –1.9 |
| 1226 | NPACT00009 | –3.5 | 1276 | NPACT01548 | –2.8 | 1326 | NPACT01552 | –1.8 |
| 1227 | NPACT00369 | –3.5 | 1277 | NPACT00381 | –2.8 | 1327 | NPACT00015 | –1.7 |
| 1228 | NPACT00889 | –3.5 | 1278 | NPACT00047 | –2.8 | 1328 | NPACT00630 | –1.7 |
| 1229 | NPACT00744 | –3.4 | 1279 | NPACT00646 | –2.8 | 1329 | NPACT00136 | –1.7 |
| 1230 | NPACT00004 | –3.4 | 1280 | NPACT00157 | –2.8 | 1330 | NPACT01005 | –1.6 |
| 1231 | NPACT00074 | –3.4 | 1281 | NPACT00374 | –2.8 | 1331 | NPACT01559 | –1.6 |
| 1232 | NPACT00742 | –3.4 | 1282 | NPACT00541 | –2.8 | 1332 | NPACT00140 | –1.6 |
| 1233 | NPACT00109 | –3.4 | 1283 | NPACT00624 | –2.8 | 1333 | NPACT00616 | –1.6 |
| 1234 | NPACT00357 | –3.4 | 1284 | NPACT01312 | –2.8 | 1334 | NPACT00014 | –1.5 |
| 1235 | NPACT01309 | –3.4 | 1285 | NPACT00010 | –2.7 | 1335 | NPACT00427 | –1.5 |
| 1236 | NPACT00134 | –3.4 | 1286 | NPACT00292 | –2.7 | 1336 | NPACT00119 | –1.5 |
| 1237 | NPACT00274 | –3.4 | 1287 | NPACT00263 | –2.7 | 1337 | NPACT00241 | –1.5 |
| 1238 | NPACT01189 | –3.4 | 1288 | NPACT00294 | –2.7 | 1338 | NPACT01007 | –1.5 |
| 1239 | NPACT00083 | –3.3 | 1289 | NPACT00296 | –2.7 | 1339 | NPACT01096 | –1.5 |
| 1240 | NPACT01252 | –3.3 | 1290 | NPACT01123 | –2.7 | 1340 | NPACT01550 | –1.5 |
| 1241 | NPACT00130 | –3.3 | 1291 | NPACT01042 | –2.7 | 1341 | NPACT00141 | –1.4 |
| 1242 | NPACT00459 | –3.3 | 1292 | NPACT00505 | –2.7 | 1342 | NPACT00016 | –1.4 |
| 1243 | NPACT00782 | –3.3 | 1293 | NPACT00762 | –2.6 | 1343 | NPACT00936 | –1.4 |

**Table S1.** *Continued*.

| **No.** | **Compound Code** | **Docking Score (kcal/mol)** | **No.** | **Compound Code** | **Docking Score (kcal/mol)** | **No.** | **Compound Code** | **Docking Score (kcal/mol)** |
| --- | --- | --- | --- | --- | --- | --- | --- | --- |
| 1344 | NPACT00657 | –1.4 | 1399 | NPACT00063 | –1.3 | 1449 | NPACT00417 | –1.2 |
| 1345 | NPACT01465 | –1.4 | 1400 | NPACT00071 | –1.3 | 1450 | NPACT00435 | –1.2 |
| 1346 | NPACT00139 | –1.4 | 1401 | NPACT00096 | –1.3 | 1451 | NPACT00439 | –1.2 |
| 1347 | NPACT01232 | –1.4 | 1402 | NPACT00099 | –1.3 | 1452 | NPACT00440 | –1.2 |
| 1348 | NPACT01562 | –1.4 | 1403 | NPACT00100 | –1.3 | 1453 | NPACT00441 | –1.2 |
| 1349 | NPACT01547 | –1.4 | 1404 | NPACT00142 | –1.3 | 1454 | NPACT00455 | –1.2 |
| 1350 | NPACT01153 | –1.4 | 1405 | NPACT00148 | –1.3 | 1455 | NPACT00465 | –1.2 |
| 1351 | NPACT00619 | –1.4 | 1406 | NPACT00155 | –1.3 | 1456 | NPACT00467 | –1.2 |
| 1352 | NPACT01557 | –1.4 | 1407 | NPACT00160 | –1.3 | 1457 | NPACT00473 | –1.2 |
| 1353 | NPACT00264 | –1.4 | 1408 | NPACT00164 | –1.3 | 1458 | NPACT00476 | –1.2 |
| 1354 | NPACT00138 | –1.4 | 1409 | NPACT00178 | –1.3 | 1459 | NPACT00489 | –1.2 |
| 1355 | NPACT00875 | –1.4 | 1410 | NPACT00180 | –1.3 | 1460 | NPACT00493 | –1.2 |
| 1356 | NPACT00011 | –1.4 | 1411 | NPACT00199 | –1.3 | 1461 | NPACT00496 | –1.2 |
| 1357 | NPACT00137 | –1.4 | 1412 | NPACT00200 | –1.3 | 1462 | NPACT00508 | –1.2 |
| 1358 | NPACT00427 | –1.4 | 1413 | NPACT00201 | –1.3 | 1463 | NPACT00516 | –1.2 |
| 1359 | NPACT00418 | –1.4 | 1414 | NPACT00202 | –1.3 | 1464 | NPACT00522 | –1.2 |
| 1360 | NPACT01558 | –1.4 | 1415 | NPACT00206 | –1.3 | 1465 | NPACT00523 | –1.2 |
| 1361 | NPACT00145 | –1.4 | 1416 | NPACT00207 | –1.3 | 1466 | NPACT00528 | –1.2 |
| 1362 | NPACT01014 | –1.4 | 1417 | NPACT00209 | –1.3 | 1467 | NPACT00529 | –1.2 |
| 1363 | NPACT00013 | –1.4 | 1418 | NPACT00214 | –1.3 | 1468 | NPACT00530 | –1.2 |
| 1364 | NPACT00073 | –1.4 | 1419 | NPACT00216 | –1.3 | 1469 | NPACT00531 | –1.2 |
| 1365 | NPACT00079 | –1.4 | 1420 | NPACT00219 | –1.3 | 1470 | NPACT00532 | –1.2 |
| 1366 | NPACT00081 | –1.4 | 1421 | NPACT00223 | –1.3 | 1471 | NPACT00533 | –1.2 |
| 1367 | NPACT00144 | –1.4 | 1422 | NPACT00228 | –1.3 | 1472 | NPACT00534 | –1.2 |
| 1368 | NPACT00437 | –1.4 | 1423 | NPACT00235 | –1.3 | 1473 | NPACT00535 | –1.2 |
| 1369 | NPACT00438 | –1.4 | 1424 | NPACT00243 | –1.3 | 1474 | NPACT00537 | –1.2 |
| 1370 | NPACT00444 | –1.4 | 1425 | NPACT00247 | –1.3 | 1475 | NPACT00539 | –1.2 |
| 1371 | NPACT00618 | –1.4 | 1426 | NPACT00249 | –1.3 | 1476 | NPACT00540 | –1.2 |
| 1372 | NPACT00653 | –1.4 | 1427 | NPACT00251 | –1.3 | 1477 | NPACT00543 | –1.2 |
| 1373 | NPACT00655 | –1.4 | 1428 | NPACT00253 | –1.3 | 1478 | NPACT00544 | –1.2 |
| 1374 | NPACT00728 | –1.4 | 1429 | NPACT00262 | –1.3 | 1479 | NPACT00545 | –1.2 |
| 1375 | NPACT00731 | –1.4 | 1430 | NPACT00266 | –1.3 | 1480 | NPACT00570 | –1.2 |
| 1376 | NPACT00968 | –1.4 | 1431 | NPACT00268 | –1.3 | 1481 | NPACT00573 | –1.2 |
| 1377 | NPACT01002 | –1.4 | 1432 | NPACT00269 | –1.3 | 1482 | NPACT00575 | –1.2 |
| 1378 | NPACT01016 | –1.4 | 1433 | NPACT00291 | –1.3 | 1483 | NPACT00595 | –1.2 |
| 1379 | NPACT01031 | –1.4 | 1434 | NPACT00318 | –1.3 | 1484 | NPACT00598 | –1.2 |
| 1380 | NPACT01457 | –1.4 | 1435 | NPACT00319 | –1.3 | 1485 | NPACT00613 | –1.2 |
| 1381 | NPACT01533 | –1.4 | 1436 | NPACT00330 | –1.3 | 1486 | NPACT00631 | –1.2 |
| 1382 | NPACT01545 | –1.4 | 1437 | NPACT00340 | –1.3 | 1487 | NPACT00632 | –1.2 |
| 1383 | NPACT01549 | –1.4 | 1438 | NPACT00355 | –1.3 | 1488 | NPACT00633 | –1.2 |
| 1384 | NPACT01551 | –1.4 | 1439 | NPACT00371 | –1.3 | 1489 | NPACT00649 | –1.2 |
| 1385 | NPACT01560 | –1.4 | 1440 | NPACT00379 | –1.3 | 1490 | NPACT00650 | –1.2 |
| 1386 | NPACT01561 | –1.4 | 1441 | NPACT00386 | –1.3 | 1491 | NPACT00665 | –1.2 |
| 1387 | NPACT01564 | –1.4 | 1442 | NPACT00393 | –1.3 | 1492 | NPACT00672 | –1.2 |
| 1388 | NPACT00023 | –1.3 | 1443 | NPACT00394 | –1.3 | 1493 | NPACT00673 | –1.2 |
| 1389 | NPACT00024 | –1.3 | 1444 | NPACT00395 | –1.3 | 1494 | NPACT00681 | –1.2 |
| 1390 | NPACT00033 | –1.3 | 1445 | NPACT00402 | –1.3 | 1495 | NPACT00696 | –1.2 |
| 1391 | NPACT00044 | –1.3 | 1446 | NPACT00404 | –1.3 | 1496 | NPACT00698 | –1.2 |
| 1392 | NPACT00046 | –1.3 | 1447 | NPACT00408 | –1.3 | 1497 | NPACT00707 | –1.2 |
| 1393 | NPACT00050 | –1.3 | 1448 | NPACT00416 | –1.3 | 1498 | NPACT00717 | –1.2 |

**Table S1.** *Continued*.

| **No.** | **Compound Code** | **Docking Score (kcal/mol)** | **No.** | **Compound Code** | **Docking Score (kcal/mol)** | **No.** | **Compound Code** | **Docking Score (kcal/mol)** |
| --- | --- | --- | --- | --- | --- | --- | --- | --- |
| 1499 | NPACT00718 | –1.1 | 1504 | NPACT00745 | –1.1 | 1508 | NPACT00771 | –1.1 |
| 1500 | NPACT00719 | –1.1 | 1505 | NPACT00746 | –1.1 | 1509 | NPACT00776 | –1.1 |
| 1501 | NPACT00720 | –1.1 | 1506 | NPACT00765 | –1.1 | 1510 | NPACT00780 | –1.1 |
| 1502 | NPACT00723 | –1.1 | 1507 | NPACT00770 | –1.1 | 1511 | NPACT00781 | –1.1 |
| 1503 | NPACT00738 | –1.1 |  |  |  |  |  |  |

**Table S2.** Estimated quick and expensive binding scores of the promising 65 NPACT compounds towards EBNA1.

| No. | **Compound Code** | **Docking Score (kcal/mol)** | |
| --- | --- | --- | --- |
|  |  | **Quick** | **Expensive** |
|  | **KWG** | **–7.8** | **–7.8** |
| 1 | NPACT01468 | −8.0 | −9.7 |
| 2 | NPACT00148 | −9.7 | −9.5 |
| 3 | NPACT00124 | −9.3 | −9.3 |
| 4 | NPACT00774 | −8.9 | −9.2 |
| 5 | NPACT01326 | −9.2 | −9.1 |
| 6 | NPACT01325 | −9.0 | −9.0 |
| 7 | NPACT00309 | −9.0 | −9.0 |
| 8 | NPACT01034 | −8.6 | −9.0 |
| 9 | NPACT01327 | −9.0 | −8.8 |
| 10 | NPACT01270 | −8.4 | −8.8 |
| 11 | NPACT00382 | −8.7 | −8.7 |
| 12 | NPACT01268 | −8.6 | −8.6 |
| 13 | NPACT00307 | −8.6 | −8.6 |
| 14 | NPACT00306 | −8.6 | −8.5 |
| 15 | NPACT00189 | −8.4 | −8.5 |
| 16 | NPACT01496 | −8.5 | −8.4 |
| 17 | NPACT01197 | −8.2 | −8.4 |
| 18 | NPACT01273 | −8.0 | −8.4 |
| 19 | NPACT00123 | −8.3 | −8.4 |
| 20 | NPACT01271 | −8.2 | −8.4 |
| 21 | NPACT01342 | −8.4 | −8.4 |
| 22 | NPACT01155 | −8.4 | −8.4 |
| 23 | NPACT01000 | −8.2 | −8.4 |
| 24 | NPACT00305 | −8.3 | −8.4 |
| 25 | NPACT01200 | −8.3 | −8.4 |
| 26 | NPACT00017 | −8.3 | −8.3 |
| 27 | NPACT00829 | −8.0 | −8.3 |
| 28 | NPACT00304 | −8.2 | −8.3 |
| 29 | NPACT00446 | −8.2 | −8.3 |
| 30 | NPACT00062 | −8.3 | −8.3 |
| 31 | NPACT01049 | −8.2 | −8.3 |
| 32 | NPACT01226 | −8.2 | −8.2 |
| 33 | NPACT00475 | −8.1 | −8.2 |
| 34 | NPACT00995 | −8.2 | −8.2 |
| 35 | NPACT00909 | −8.0 | −8.2 |
| 36 | NPACT00700 | −8.1 | −8.1 |
| 37 | NPACT00576 | −7.9 | −8.1 |
| 38 | NPACT00560 | −8.1 | −8.1 |
| 39 | NPACT00195 | −8.0 | −8.0 |
| 40 | NPACT00034 | −7.9 | −8.0 |
| 41 | NPACT01483 | −7.9 | −7.9 |
| 42 | NPACT01248 | −7.8 | −7.9 |
| 43 | NPACT00558 | −7.9 | −7.9 |
| 44 | NPACT01125 | −7.9 | −7.9 |
| 45 | NPACT01427 | −7.9 | −7.9 |
| 46 | NPACT00549 | −7.9 | −7.9 |
| 47 | NPACT01341 | −7.9 | −7.9 |
| 48 | NPACT01209 | −7.8 | −7.8 |
| 49 | NPACT00289 | −7.8 | −7.8 |

**Table S2.** *Continued.*

| No. | **Compound Code** | **Docking Score (kcal/mol)** | |
| --- | --- | --- | --- |
|  |  | **Quick** | **Expensive** |
| 50 | NPACT00176 | −7.8 | −7.8 |
| 51 | NPACT00075 | −7.8 | −7.8 |
| 52 | NPACT01154 | −7.8 | −7.8 |
| 53 | NPACT00557 | −8.1 | −7.8 |
| 54 | NPACT01329 | −7.8 | −7.8 |
| 55 | NPACT01260 | −7.9 | −7.8 |
| 56 | NPACT00472 | −7.9 | −7.8 |
| 57 | NPACT01328 | −8.2 | −7.8 |
| 58 | NPACT00512 | −8.3 | −7.8 |
| 59 | NPACT01410 | –8.4 | −7.8 |
| 60 | NPACT00864 | –8.5 | −7.8 |
| 61 | NPACT00447 | –8.5 | −7.8 |
| 62 | NPACT01235 | –8.5 | −7.7 |
| 63 | NPACT01316 | –8.7 | −7.7 |
| 64 | NPACT00685 | –8.7 | −7.6 |
| 65 | NPACT01033 | –9.3 | −7.6 |

^a^ Data sorted based on the expensive binding scores.

**Table S3.** The anticipated quick and expensive binding scores and MM/GBSA binding energies (in kcal/mol) over 5 ns for the top 55 NPACT compounds and KWG towards EBNA1 ^a^.

| No. | **Compound Code** | **Docking Score (kcal/mol)** | | **MM/GBSA Binding Energy (kcal/mol)** |
| --- | --- | --- | --- | --- |
|  |  | **Quick** | **Expensive** |  |
|  | **KWG** | **–7.8** | **–7.8** | **–33.5** |
| 1 | NPACT01468 | −8.0 | −9.7 | −38.0 |
| 2 | NPACT01270 | −8.4 | −8.8 | −33.0 |
| 3 | NPACT00176 | −7.8 | −7.8 | −31.8 |
| 4 | NPACT01200 | −8.3 | −8.4 | −31.7 |
| 5 | NPACT01273 | −8.0 | −8.4 | −31.4 |
| 6 | NPACT00195 | −8.0 | −8.0 | −30.9 |
| 7 | NPACT00382 | −8.7 | −8.7 | −30.8 |
| 8 | NPACT00557 | −8.1 | −7.8 | −29.8 |
| 9 | NPACT00189 | −8.4 | −8.5 | −29.6 |
| 10 | NPACT01496 | −8.5 | −8.4 | −29.6 |
| 11 | NPACT01154 | −7.8 | −7.8 | −29.2 |
| 12 | NPACT00560 | −8.1 | −8.1 | −28.5 |
| 13 | NPACT01248 | −7.8 | −7.9 | −28.5 |
| 14 | NPACT00475 | −8.1 | −8.2 | −28.4 |
| 15 | NPACT00549 | −7.9 | −7.9 | −28.3 |
| 16 | NPACT00034 | −7.9 | −8.0 | −28.2 |
| 17 | NPACT00558 | −7.9 | −7.9 | −28.1 |
| 18 | NPACT00576 | −7.9 | −8.1 | −27.6 |
| 19 | NPACT00148 | −9.7 | −9.5 | −26.6 |
| 20 | NPACT00304 | −8.2 | −8.3 | −26.4 |
| 21 | NPACT00446 | −8.2 | −8.3 | −26.3 |
| 22 | NPACT00307 | −8.6 | −8.6 | −26.1 |
| 23 | NPACT01209 | −7.8 | −7.8 | −25.5 |
| 24 | NPACT01325 | −9.0 | −9.0 | −25.4 |
| 25 | NPACT01268 | −8.6 | −8.6 | −24.7 |
| 26 | NPACT00062 | −8.3 | −8.3 | −24.7 |
| 27 | NPACT01049 | −8.2 | −8.3 | −24.7 |
| 28 | NPACT01327 | −9.0 | −8.8 | −24.3 |
| 29 | NPACT01342 | −8.4 | −8.4 | −24.2 |
| 30 | NPACT00829 | −8.0 | −8.3 | −24.2 |
| 31 | NPACT00774 | −8.9 | −9.2 | −24.0 |
| 32 | NPACT01197 | −8.2 | −8.4 | −23.1 |
| 33 | NPACT00309 | −9.0 | −9.0 | −23.0 |
| 34 | NPACT01155 | −8.4 | −8.4 | −23.0 |
| 35 | NPACT00305 | −8.3 | −8.4 | −22.9 |
| 36 | NPACT00124 | −9.3 | −9.3 | −22.8 |
| 37 | NPACT01271 | −8.2 | −8.4 | −21.9 |
| 38 | NPACT00306 | −8.6 | −8.5 | −21.8 |
| 39 | NPACT00700 | −8.1 | −8.1 | −21.6 |
| 40 | NPACT01125 | −7.9 | −7.9 | −20.8 |
| 41 | NPACT00017 | −8.3 | −8.3 | −19.5 |
| 42 | NPACT00995 | −8.2 | −8.2 | −18.6 |
| 43 | NPACT00909 | −8.0 | −8.2 | −18.6 |
| 44 | NPACT00075 | −7.8 | −7.8 | −18.1 |
| 45 | NPACT01034 | −8.6 | −9.0 | −17.3 |
| 46 | NPACT01226 | −8.2 | −8.2 | −16.2 |
| 47 | NPACT00289 | −7.8 | −7.8 | −14.6 |
| 48 | NPACT00123 | −8.3 | −8.4 | −14.5 |
| 49 | NPACT01000 | −8.2 | −8.4 | −14.0 |

**Table S3.** *Continued.*

| No. | **Compound Code** | **Docking Score (kcal/mol)** | | **MM/GBSA Binding Energy (kcal/mol)** |
| --- | --- | --- | --- | --- |
|  |  | **Quick** | **Expensive** |  |
| 50 | NPACT01329 | −7.8 | −7.8 | −14.0 |
| 51 | NPACT01341 | −7.9 | −7.9 | −13.4 |
| 52 | NPACT01260 | −7.9 | −7.8 | −13.1 |
| 53 | NPACT01483 | −7.9 | −7.9 | −9.6 |
| 54 | NPACT01326 | −9.2 | −9.1 | −7.4 |
| 55 | NPACT01427 | −7.9 | −7.9 | −6.9 |

^a^ Data were arranged according to the MM/GBSA binding energy over 5 ns.
